# Cross-linguistic conditions on word length

**DOI:** 10.1371/journal.pone.0281041

**Published:** 2023-01-27

**Authors:** Søren Wichmann, Eric W. Holman

**Affiliations:** 1 Cluster of Excellence ROOTS, Kiel University, Kiel, Germany; 2 Department of Psychology, University of California, Los Angeles, Los Angeles, California, United States of America; Universidad Complutense Madrid, SPAIN

## Abstract

Based on a dataset representing close to ¾ of the world’s languages we investigate differences among languages and between items on the Swadesh list with regard to mean word length from a linguistic typological point of view. Mapping the world-wide distribution of word length shows convergence at a continent-wide level, a Pacific Rim signature, and a tendency for large word length averages to be a recessive trait. The amount of data, which is unparalleled in previous, related studies, allows us to provide more solid estimates and accounts for the interrelationships between word length, phoneme segment inventory size, and population size than was previously possible. Word length differences between items exhibit robust, universal tendencies, which are discussed in relation to other quantities, including stability, synonymy, and attestation.

## Introduction

In this paper we present basic statistics on word length across languages of the world and investigate linguistic variables that determine the variability in word length, such as inheritance, areal influence, phoneme inventory size, and parts of speech membership. For our purposes, a word is defined as an entry in a dictionary which is marked as a single, separate string by leading and trailing spaces and provides a translational equivalent of a specific concept commonly lexicalized throughout the languages of the world. We use a selection of words expressing the meanings on the 100-item Swadesh list [1] and data from the ASJP database, which currently [2] contains 100-item lists from 1267 different languages as defined by the ISO 639–3 standard and also lists from an additional 4114 languages that only cover the 40-item subset of the Swadesh list defined in [3]. Altogether the data represents around 73% of the world’s languages, a vast expansion from the small samples of large languages typical in previous research on word length.

Cognitive capacities that presumably do not vary among human societies, even if there may be some variation across individuals, most certainly induce constraints on the length of phonological strings representing units of speech. For instance, word length has an effect on the number of different words that subjects can retain in working memory [4]. Here, however, we will be concerned with phenomena related to word length that are specific either to languages or to areas where the languages are spoken. Thus, our approach pertains to the field of linguistic typology. Typology is traditionally concerned with the mapping of differences and similarities across languages as well as interrelationships among structures [5], and it tends to be driven more by empirical observations than by theories and models [6]. Moreover, it increasingly pays attention to the larger historical picture of language evolution during the past several millennia in a realization that “(i) typological distributions are historically grown and (ii) that they are interrelated with other distributions” [7].

With regard to the areal factor, we do not look for specific geographical or ecological influences on word length. The one nonlinguistic factor in our purview is population size, since previous research has suggested that there is a negative correlation between word length and population sizes, indicating a tendency for larger populations to have shorter words [8]. Our data is presented in such a way that it can readily be incorporated into other linguistic databases, such as WALS [9], and in general be used in further investigations of possible correlates and implications of word length.

### Research questions

This subsection presents the research questions that we have found productive to pursue. Each question is listed here in the same order as the subsections of the section ‘Results’, where our findings will be presented. Questions 1–2 concern differences between languages as regards mean word length across items; questions 3–7 concern differences between items on the word list; questions 8–9 refer to word length in comparison to other typological features.

1. *Inheritance and areality*. How does mean word length across the 40 ASJP items differ among phylogenetic groups and geographic areas?
2. *Word length*, *phoneme inventory and population sizes*. Correlations in previous literature have suggested interrelationships between these three variables. Can further inferences be drawn from the correlations?
3. *Differences between items*. Are there differences in word length for different items on the 100-item list?
4. *Stability of items*. Does word length of individual items across languages relate to stability as measured by the tendency for words to retain similar shapes across related languages?
5. *Synonyms*. Does word length of items relate to the proportion of languages in which an item is represented by two or more synonymous words?
6. *Attestation*. Is there a relation between the frequency with which items are attested in the database and word length?
7. *Semantics-syntax*. Are there cross-linguistic tendencies for words belonging to different semantic classes commonly implying different morphosyntactic behaviors—i.e., words denoting (a) things, (b) actions, (c) properties and (d) function words—to differ in word length, and are these possible differences also related to stability?
8. *Morphology*. To what degree do systematic, cross-linguistic morphological patterns determine variation in mean word length across languages (cf. questions 1–2) and between items (cf. question 3)?
9. *Stability of word length*. How stable is word length as compared to other typological features of languages?

## Materials and methods

### Data preparation

Several analyses presented in this paper aggregate over units such as parts of speech, groups of languages, areas, etc. Ultimately these aggregates are based on the *minimal simple unit of analysis*, which is the average length of (a maximum of two) synonyms for a concept in a doculect. A doculect is a language variety as defined by a particular source. Even if different sources describe one and the same variety, the data would be considered as belong to different doculects. After selecting the single form or the two synonyms potentially feeding into the minimal simple unit, phrases (anything with one or more spaces in it), are removed from consideration. For languages that obligatorily require some forms to be inflected, the practice in developing the ASJP database has been to strip forms of their inflectional affixes. This means that the term ‘word length’ used here is somewhat imprecise—perhaps ‘stem length’ would have been more adequate. Words (in the sense just described) in the ASJP database are transcribed using ASJPcode [10]. While this transcription system merges phonemes into classes of phonemes, it does not alter the number of phonemes in words. ASJPcode is equipped with modifiers that indicate cases where two or three symbols together represent a single phoneme, and these modifiers are interpreted in their intended functions for the present purposes of counting word length. The modifiers indicating consonant glottalization (”) and vowel nasalization (*) are ignored because they do not alter word length. The modifier indicating loanword status (%) is also ignored.

The average of these minimal simple units of analysis across words from different doculects pertaining to a language yields the *minimal aggregate unit of analysis*, which is used in all analyses. For the purposes of analyses presented in this paper, this unit is defined by doculects sharing the same ISO 639–3 code. If no ISO-code could be assigned to a doculect, a doculect is treated as a unique ISO 639–3 representative and a pseudo-code is constructed from its name in ASJP or, when available, an ISO 639–3 pseudo-code provided by the ASJP database is used (such pseudo-codes consist of two letters and a 0, and are provided when the editors of ASJP felt that an ISO 639–3 would be appropriate, but was not available). At https://doi.org/10.5281/zenodo.6344023 we also present a table (Data-02 WALS data.txt) with data appropriate for the inclusion in WALS [9]. Here the minimal aggregate unit of analysis is an average over words in doculects pertaining to one and the same WALS code. For calculating a representative mean word length of a language, we average word length counts (the minimal aggregate unit of analysis) across the 40-item subset of the Swadesh list [3] which forms the core of the database.

The data used in the analyses of this paper is collected in a file called Data-01 ASJP data raw.txt, available at https://doi.org/10.5281/zenodo.6344023. It contains columns for ISO 639–3 codes, doculect names, language codes and family classification from WALS [9], Glottolog [11] family classification, coordinates, population figures, word length for each item, a percentage of doculects with two or more synonyms, a percentage for the total number of attestations among the 40 items, a percentage for the total number of attestations among the 100 Swadesh items [1], word length averaged over, respectively, the 40 ASJP items and the full Swadesh list, and assignments of ‘area’, ‘continent’, and ‘macrocontinent’ from Autotyp [12]. More detail on data preparation is given in S1 File.

### Measuring geographical distances

For looking at differences in word length between languages as a function of the geographical distance between them, we apply both the Great Circle Distance, which is computed using the standard formula (e.g. as found in [13]), and an approximation to a walking distance. The approximate walking distance is the one called DD-0.25 in [14]. Its designation, which can be read ‘Delaunay-Dijkstra one quarter degree’, is descriptive of how it works. Paths are constrained to waypoints, which are all currently populated places found in the database at http://www.geonames.org/. One populated place per cell in a one quarter-degree grid was chosen randomly, yielding 133,769 waypoints. The waypoints were submitted to Delaunay triangulation [15], and the walking distance represents the shortest path, as found by Dijkstra’s algorithm [16], through this network, using the Great Circle Distance for steps between waypoints. Thus, this walking distance uses a selection of today’s populated places as a proxy for stations admitting passage by foot.

### Point locations representing genera

For the purpose of reducing the extension of a genus [17] to a manageable single location, we have chosen as a representative location that of the language within the genus having the smallest average distance to the other languages in the genus (with the choice being made arbitrarily when only two languages were available or in other cases of ties). This minimal-distance location has been shown to give a surprisingly good approximation to a language family homeland [18].

### Scaling of variables for phoneme inventory size and population size

Following common practice, population size is here transformed logarithmically to produce an approximately normal distribution. Phoneme inventory size is also transformed logarithmically for the same reason (histograms are offered in S2 File). The distribution of mean word length is already approximately normal. All three variables are then scaled by subtracting the mean and dividing by the standard deviation.

### Sorting languages into macroareas

**Fig 1:**
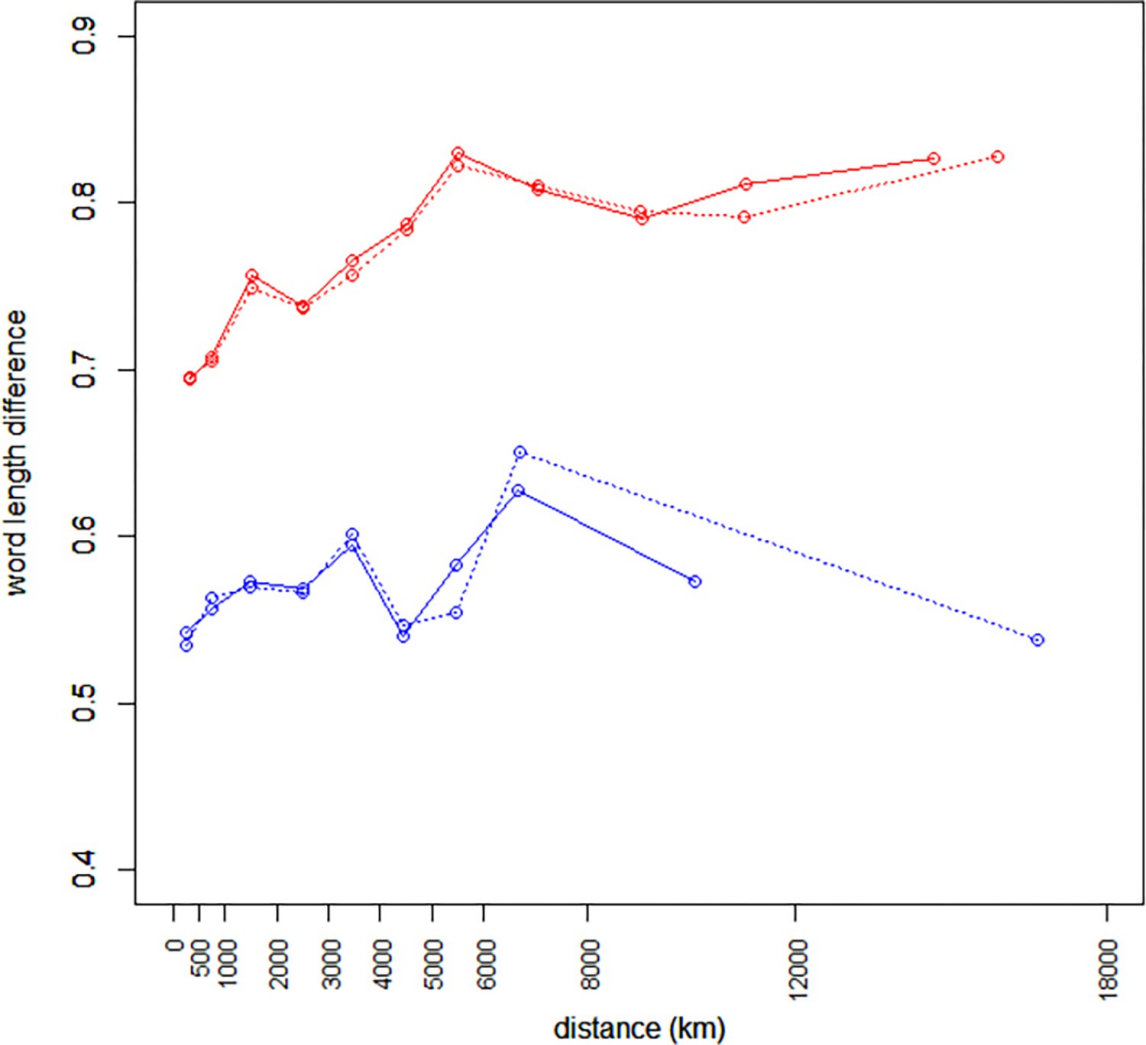
Word length difference as a function of geographical distance for unrelated (red lines) and unrelated (blue lines) languages, using the Great Circle Distance (solid lines) and an approximate walking distance (dotted lines). For both variables the means within bins are plotted.

For analyzing relationship between the variables inventory size, population size, and word length, the relevant languages are sorted into six macroareas, which are the same as the ‘continents’ defined in Autotyp [12], except for two mergers: C America, E N America, and W N America are combined into N America; and N-C Asia, S/SE Asia, and W and SW Eurasia are combined into Eurasia. The use of macroareas is motivated by our finding that geographical proximity has an effect on word length similarity within a range of some 5000 km (Fig 1).

### The two-hemisphere test

In order to test whether word length differences between items on the Swadesh list are robust across language families and areas, we apply the same test that we used in a study of the differential stabilities of typological features [19]. The test requires sorting the data into two sets that are independent of one another as far as genealogical and areal effects are concerned. The sets are located respectively in the Eastern and Western Hemispheres, with each family assigned to at most one hemisphere. Since it has been found [20] that the approximate limit of diffusion of structural features is around 5000 km, similarly to what was observed for word length in the present paper, we exclude some families altogether in order to widen the gap correspondingly between the hemispheres. Thus, Eskimo-Aleut is excluded from the Western Hemisphere, and Altaic, Ainu, Chukotko-Kamchatkan, Japanese, Korean, Nivkh, Yeniseian, and Yukaghir are excluded from the Eastern Hemisphere. This procedure is intended to ensure that any historical relationship between the hemispheres, whether by inheritance or diffusion, is too remote to have a discernable influence on the properties of their languages.

### Stability

We define the stability of a meaning or item on the Swadesh list in terms of a similarity measure for word pairs which is the counterpart of the similarity measure used in many papers drawing upon the ASJP database since the publication of Ref. [21], thus here referred to as ASJP similarity. ASJP similarity is based ultimately on the Levenshtein distance (LD), which for any two words is defined as the minimum number of changes (insertions, deletions, or substitutions) of individual symbols sufficient to change one word into the other. Following other scholars [22, 23], LD is then divided by the length of the longer word to produce normalized LD (LDN). To measure the difference between two languages, LDN is calculated between pairs of words with the same meaning in the two languages and then averaged across all of the ASJP meanings that are attested in both languages. This average is finally divided by a baseline consisting of the average LDN between all pairs of words with different ASJP meanings attested in the two languages; the quotient is called LDND (LDN divided). See Ref. [24] for more justification of the final division. ASJP similarity is defined as 1 –LDND.

Now, to estimate the stability of a meaning, LDND between two languages is calculated as just described, except separately for each meaning rather than averaged across meanings. With meaning still held constant, LDND is next averaged across all language pairs in the same WALS genus and then averaged across genera. This average reflects the changes that have accumulated in the words for the given meaning since the language pairs within genera diverged from their common ancestors. Stability of the meaning is therefore estimated by the average similarity, 1 –LDND. Synonyms, phrases, and multiple lists per ISO code are treated as described in S1 File.

Genera are particularly suited for the estimation of stability because their definition includes a time depth, 3500–4000 years. Since similarity depends on time depth as well as on stability, holding time depth approximately constant reduces variability in the estimate of stability. The estimate is expected to be most accurate for relatively stable meanings, such as the 100 Swadesh items, because words for less stable meanings will undergo repeated changes and their estimated stability will approach zero. Stability as defined here includes resistance to borrowing along with resistance to internal changes in sound or meaning.

The present estimate of the stability of meanings is related to an earlier measure of stability for structural features [19]. S3 File describes this relationship.

## Results

### Inheritance and areality

**Fig 2:**
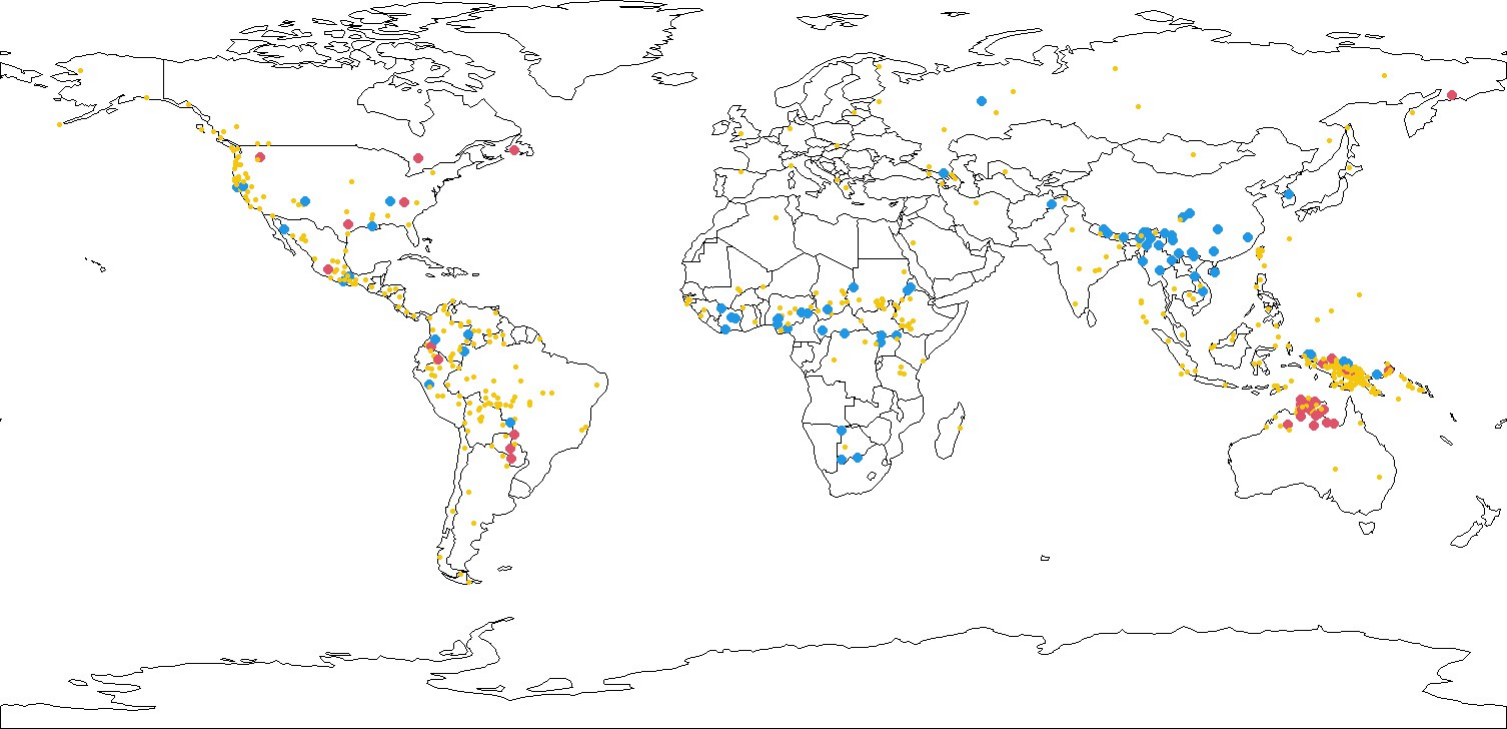
Large (red dots), small (blue dots), and intermediate (yellow dots) values of mean word length of WALS genera. Country borders were generated using [27].

Is mean word length an inherited or a diffused characteristic? The unsurprising answer is that it is both. This is easily shown through the plots in Figs 1 and 2 offered below. As for any typological feature, making a quantitative comparison of the exact contribution of each of the two factors is fraught with difficulties [25]. One of main problems is that phylogenetic distance is not unrelated to geographical distance, given that more closely related languages tend to be spoken closer to one another than more distantly related languages. So we will not attempt to quantify the respective contributions of inheritance and areality to mean word length.

Ref. [20] plotted differences in structural features as a function of geographical distances separately for pairs of related and unrelated languages. In Fig 1 we apply the same approach to absolute differences in word length for all pairs involving 5004 languages in our database (cf. S1 File). Languages are defined as related if they belong to the same family according to Glottolog. Just as in [20], we group geographical distances into bins with the following lower limits (in km): 0, 500, 1000, 2000, 3000, 4000, 5000, 6000, and 8000 for both related and unrelated languages; for unrelated languages subsequent limits are 10000 and 12000, while for related languages we continue with a single 8000+ bin. The higher resolutions at smaller distances and lower resolutions at higher distances serve to make sample sizes less uneven. Unlike [20] we not only plot geographical distances as measured by the Great Circle Distance, but also an approximation to walking distances [14], cf. Materials and Methods.

Fig 1 shows that, except for unexplained bumps at the 1000–2000 km bin, both pairs of curves rise steadily until the 3000–4000 km bin. Hereafter the (lower) curves for related languages enter into fluctuation, while the (upper) curves for unrelated languages continue to rise until the 5000–6000 km bin, after which they also fluctuate. We made similar plots using family definitions from WALS (shown in S4 File). These curves were barely distinguishable from those using Glottolog family definitions. This suggests that future updates to either classification are unlikely to change the results appreciably. It is only for the largest distances that the Great Circle Distance and the walking distance approximation yield markedly different results, which serves to underscore the robustness of the results.

The behavior of Fig 1 is qualitatively very similar to that of the pool of typological features plotted in Fig 1 of Ref. [20], the main differences being that for the pool of typological features both curves are smoother and continue to rise for another 1000 km or so. It is clear, then, that word length shows the typical behavior of a typological feature: it preserves a strong genealogical signal which, on average, tells apart related from unrelated language pairs at any degree of geographical proximity, and at the same time it shows that similarity depends on geography as well—a dependency which is strong enough to persist even at a continent-sized range of around 5000 km.

In order to offer more specific information about the areal tendencies we provide the map in Fig 2. Each dot represents mean word length of a WALS genus. Large blue dots represent values that are smaller than 3.5 (‘very short’ or ‘extremely short’ in our WALS-styled categorical distinctions in S1 File), large red dots represent values greater than 5.5 (‘very long’ or ‘extremely long’), and small yellow dots represent intermediate values. Representative point locations for the genera have been selected as described in Materials and Methods. One of Dryer’s criteria for a genus was a time depth of 3500–4000 years [17]. While we do not necessarily subscribe to the accuracy of this estimate across all genera [26], we can cautiously say that averaging over genera aligns the averages with a time depth of a few thousand years.

What Fig 2 shows, then, is a diachronically conservative picture of the worldwide distribution of mean word length, with a focus on the extreme values. Interestingly, the largest values are in the Pacific Rim area [28]. A slightly more inclusive criterion for the red dots would produce more of them along the east coast of Eurasia, further consolidating the Pacific Rim distribution, although this would also cause a few red dots to show up further to the west. The smallest mean word length values are found in high concentration in SE Asia, and are scattered throughout the Americas, Sub-Saharan Africa, and northern New Guinea. If a tendency for a language to have long words is a Pacific Rim feature it must be very old, and since it is scarcely found elsewhere it must be a retention almost wherever it is found. In contrast, a tendency for short words does not show much geographic patterning, and therefore may be an innovative trait which can occasionally show up and which can develop further into an areal feature, as evidenced by the languages of SE Asia (where this trait seems to have gone hand in hand with the diffusion of tone). Reconstructing the word length feature for different families would be a way to further investigate the validity of this model.

We also plotted mean word length for the three area levels of Autotyp [12], i.e. ‘areas’ (24 categories), ‘continents’ (10 categories), and ‘macrocontinents’ (4 categories). The results (see S5 File) show that the behavior of ‘areas’ is to a large extent subsumed by that of ‘continents’, whereas the ‘macrocontinents’ collapse too much variation to be of any use. These maps are consistent with our plot in Fig 1, which showed effects that range over some 5000 km but not much beyond, and they are also in line with the general recommendation of Dryer [29] to use continent-sized areas in controls for areal effects in typological studies.

### Word length, phoneme inventory and population sizes

Previous work has suggested relationships among all of the following three variables: word length, phoneme inventory sizes, and (log of) population sizes. Nettle [30] reported a negative correlation between phoneme inventory sizes and word length; Hay & Bauer [31] reported a positive correlation between population sizes and phoneme size inventories; finally, Wichmann et al. [8] closed the circle by reporting a negative correlation between word length and population sizes. While their results are difficult to assess because of non-independence in the data, other papers have added nuances by separately looking at vowel and consonant inventory sizes in relation to word length [32] or by adding syllable complexity to the mesh of possibly inter-correlated variables [33]. The emerging picture is that we expect larger populations to have shorter words and larger inventories.

**Fig 3:**
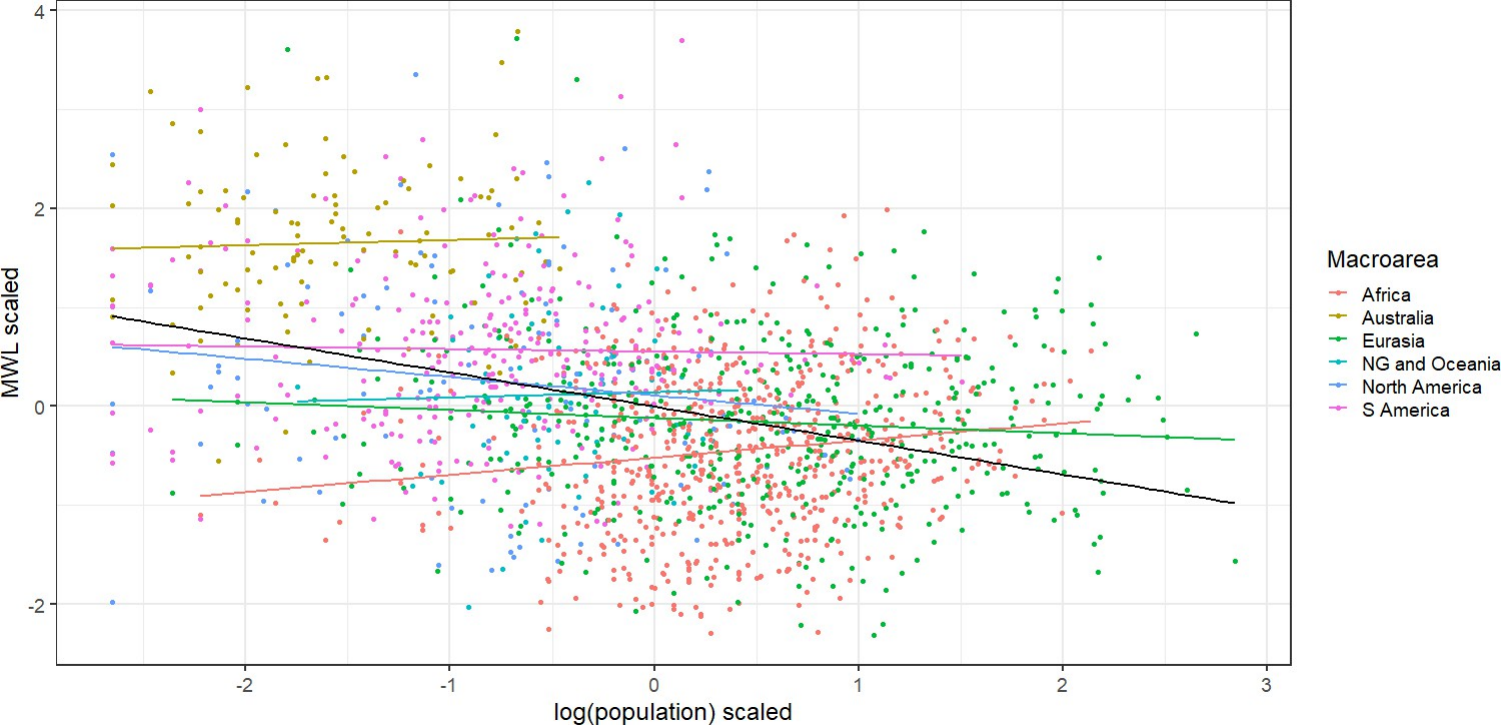
Scaled mean word length as a function of scaled log population, for 1706 languages in six macroareas. Regression lines fitted within each area are color-coded like the languages; the regression line for all 1706 languages is black.

For revisiting these relations our current database contributes the largest sample to date. Data on inventory sizes is found in PHOIBLE [34], and the overlap of their data with ASJP provides us with data from 1706 languages for the three variables. Fig 3 plots scaled mean word length against scaled log population for the 1706 languages. Since the variables are scaled with zero mean and unit standard deviation, the slope of each regression line is the same as Pearson’s *r* between the variables. The regression lines within the six macroareas all have slopes near zero, with three slightly positive and three slightly negative. S6 File provides the actual numerical values, of which the mean is 0.01. S6 File also reports regressions within the largest families; 11 coefficients are positive, 7 are negative, and the mean is 0.06. The regression line for all the languages, however, has an obviously negative slope of -0.34. This kind of mismatch between within-group and overall relationships is known as Simpson’s paradox, after [35]. In Fig 3, the overall negative correlation reflects differences between macroareas: Australia and also the Americas have small populations and long words; Africa and Eurasia have large populations and short words; and New Guinea & Oceania is intermediate.

To test within-group correlations more formally, languages are sorted into families (as defined in Glottolog) nested within macroareas. The data is then fed to a mixed effects regression model with mean word length as the dependent variable and log population and log inventory size as independent variables. Complete results are in S6 File. The regression coefficients are inconsequential: 0.08 and -0.05 for log population and log inventory size, respectively.

To complement the test of differences within groups, differences between macroareas are tested in a multivariate analysis of variance. Complete results are in S7 File. The test produces a highly significant approximate F(15,5100) = 109.97. It should be kept in mind, however, that the test assumes independence of languages within macroareas.

The question of significant differences between macroareas motivates using macroareas rather than languages as the units of analysis, which assumes that macroareas rather than languages are independent. For this purpose, a single score on each variable must be chosen to represent each macroarea; the conventional choice is the mean across the languages in a macroarea. Table 1 therefore displays the correlations of macroarea means between variables across macroareas; correlations of individual language scores across languages are also included for comparison.

**Table 1:** Pearson correlations between mean word length, log inventory, and log population. Coefficients in the lower triangle are based on macroarea means, correlated across macroareas; coefficients in the upper triangle are based on individual languages, correlated across languages.

	word length	log inventory	log population
word length		-0.37	-0.34
log inventory	-0.61		0.36
log population	-0.92	0.71	

The correlation between word length and log population across languages is the same as the slope of the overall regression line in Fig 3. The other correlations across languages are similar in strength. The much greater strength of the correlations involving macroarea means is expected because means are less variable than individual scores. More newsworthy is the fact that the negative correlation of word length with log population is significant (p < 0.01) despite being across only six macroareas. It should, however, be kept in mind that the null hypothesis for the correlations across macroareas includes the assumption that that the six macroareas are mutually independent. The correlations of log inventory with word length and log population are not significant (p = 0.20 and 0.11, respectively).

In this section our main focus has been on the relationship between population size and mean word length. Within groups such as macroareas or families, correlations tend to be small and to show fluctuating signs, whereas a large negative correlation emerges when aggregating over macroareas. This apparent paradox implies that the process through which increased population sizes come to have a significant effect on word length is not just a continuation over a longer time span of the same processes operating within macroareas and families, but rather an early process of limited duration but lasting repercussions.

The correlations involving inventory size could not be shown to be significant and are not discussed further.

### Word length differences across items

In order to address the question whether there are differences in word length for different items on the 100-item list, we first establish values for mean word length controlling for genealogical and areal effect by aggregating over WALS genera. In other words, we find the mean for each item across doculects within languages (the minimal aggregate unit of analysis), then average these values across languages within genera, and finally we average across genera. The results are displayed in Table 2, where the items are arranged by increasing mean word length values. For convenience and to facilitate replicability, the data in Table 2 is repeated along with data on stabilities in Table 3 below as well as summary data on synonymy and percent attestations discussed further on in the paper in a single sheet in S8 File. The amount of data per item ranges from 177 to 608 genera (out of 610) and from 471 to 5027 languages (out of 5226), the most incompletely attested item being ‘claw’ and the best attested being ‘water’.

**Table 2:** Mean word length for items aggregated over WALS genera.

Rank	Item	Mean	Rank	Item	Mean	Rank	Item	Mean	Rank	Item	Mean
1	I	**3.034**	26	breast	**4.195**	51	good	**4.551**	76	root	**4.825**
2	you	**3.065**	27	blood	**4.255**	52	moon	**4.551**	77	white	**4.834**
3	not	**3.283**	28	bone	**4.268**	53	two	**4.553**	78	hot	**4.864**
4	this	**3.322**	29	dog	**4.275**	54	hair	**4.563**	79	many	**4.946**
5	that	**3.523**	30	flesh	**4.289**	55	sleep	**4.568**	80	black	**4.968**
6	water	**3.526**	31	stone	**4.298**	56	bird	**4.568**	81	bark	**4.970**
7	come	**3.737**	32	earth	**4.314**	57	liver	**4.596**	82	sand	**4.977**
8	who	**3.785**	33	head	**4.317**	58	smoke	**4.601**	83	long	**4.993**
9	eat	**3.841**	34	fish	**4.327**	59	grease	**4.603**	84	heart	**5.019**
10	say	**3.848**	35	burn	**4.341**	60	kill	**4.611**	85	full	**5.025**
11	tree	**3.860**	36	belly	**4.357**	61	woman	**4.620**	86	fly	**5.026**
12	fire	**3.860**	37	path	**4.368**	62	hear	**4.648**	87	dry	**5.027**
13	what	**3.876**	38	seed	**4.393**	63	horn	**4.650**	88	red	**5.055**
14	drink	**3.877**	39	sun	**4.402**	64	ear	**4.736**	89	cloud	**5.055**
15	we	**3.962**	40	egg	**4.418**	65	big	**4.756**	90	swim	**5.094**
16	give	**4.022**	41	foot	**4.420**	66	lie	**4.764**	91	stand	**5.095**
17	name	**4.041**	42	person	**4.446**	67	man	**4.765**	92	cold	**5.101**
18	see	**4.059**	43	skin	**4.452**	68	tongue	**4.768**	93	all	**5.131**
19	hand	**4.072**	44	leaf	**4.474**	69	sit	**4.775**	94	claw	**5.302**
20	tooth	**4.098**	45	nose	**4.479**	70	ash	**4.779**	95	small	**5.337**
21	eye	**4.141**	46	tail	**4.489**	71	mountain	**4.780**	96	yellow	**5.501**
22	louse	**4.143**	47	walk	**4.513**	72	know	**4.783**	97	knee	**5.501**
23	rain	**4.158**	48	feather	**4.517**	73	new	**4.786**	98	green	**5.510**
24	mouth	**4.159**	49	one	**4.543**	74	neck	**4.796**	99	star	**5.513**
25	die	**4.187**	50	bite	**4.548**	75	night	**4.815**	100	round	**6.066**

**Table 3:** Stability of items measured as ASJP similarity aggregated over WALS genera (apparent ties are ranked according to unrounded values).

Rank	Item	Stab.	Rank	Item	Stab.	Rank	Item	Stab.	Rank	Item	Stab.
1	I	**38.08**	26	sun	**27.53**	51	come	**22.04**	76	feather	**17.36**
2	water	**36.21**	27	path	**27.29**	52	bite	**21.92**	77	neck	**17.36**
3	you	**35.07**	28	fish	**27.22**	53	grease	**21.72**	78	sand	**17.32**
4	louse	**34.56**	29	breast	**27.22**	54	egg	**21.34**	79	stand	**17.12**
5	eye	**34.26**	30	head	**26.70**	55	tail	**20.90**	80	red	**17.11**
6	name	**34.06**	31	moon	**26.43**	56	woman	**20.16**	81	green	**17.09**
7	tongue	**33.18**	32	mouth	**26.27**	57	black	**20.10**	82	say	**16.60**
8	tooth	**32.97**	33	one	**26.24**	58	this	**20.08**	83	cold	**16.35**
9	fire	**32.97**	34	knee	**26.02**	59	yellow	**20.06**	84	know	**16.21**
10	ear	**32.30**	35	skin	**25.95**	60	dry	**19.94**	85	not	**15.94**
11	stone	**31.99**	36	hear	**25.77**	61	full	**19.77**	86	round	**15.53**
12	two	**31.98**	37	rain	**25.58**	62	root	**19.76**	87	sit	**15.50**
13	bone	**31.43**	38	bird	**24.53**	63	fly	**19.76**	88	belly	**15.22**
14	nose	**31.10**	39	eat	**24.45**	64	long	**19.70**	89	kill	**15.21**
15	tree	**30.56**	40	what	**24.41**	65	see	**19.61**	90	cloud	**14.72**
16	dog	**30.27**	41	night	**24.27**	66	swim	**19.60**	91	seed	**14.52**
17	we	**30.10**	42	star	**24.17**	67	mountain	**19.47**	92	hot	**14.33**
18	hand	**29.71**	43	sleep	**23.94**	68	heart	**19.11**	93	good	**14.33**
19	blood	**29.66**	44	smoke	**23.58**	69	claw	**18.99**	94	all	**13.97**
20	die	**29.12**	45	give	**23.56**	70	white	**18.95**	95	big	**13.85**
21	horn	**28.99**	46	person	**23.40**	71	ash	**18.63**	96	bark	**13.68**
22	drink	**28.86**	47	man	**22.52**	72	hair	**18.28**	97	that	**12.83**
23	liver	**28.23**	48	who	**22.30**	73	walk	**18.06**	98	small	**12.42**
24	leaf	**28.06**	49	earth	**22.29**	74	flesh	**17.93**	99	lie	**12.18**
25	new	**27.87**	50	foot	**22.18**	75	burn	**17.64**	100	many	**12.05**

Now we apply the two-hemisphere test described in Materials and Methods. Specifically, we correlate the ranks of mean word length for items (again aggregating over WALS genera) in the two hemispheres in order to assess the degree to which these ranks are similar. The result is a Spearman’s rank correlation coefficient of ρ = 0.76. This compares favorably to the value ρ = 0.51 found earlier [14] for stabilities of typological features. The healthy correlation confirms that differences in word length for different items really are largely inherent in these items. The strength of the correlation also exemplifies a more general finding, described in S9 File, that supports aggregating over WALS genera.

In sum, there are strong, universal tendencies for specific items to be shorter or longer relative to other specific items regardless of genealogical or areal affiliation of the language in which these differences are observed. Next, we go on to look at correlates of word lengths across items, aiming to shed light on why these differences exist.

### Stability across items

We expect that items which are frequently used are both short and stable. It is well known that there is a tendency for an inverse, nonlinear relationship between word length and frequency [36–44]. Additionally, it has been observed that more frequent words tend to be less often replaced in the evolution of languages [45–47]. Both observations are based on more limited samples of languages than available for the present study. Frequency data is not included as such in the ASJP database and is not available for most of the languages in our sample, although a possible simplied measure of frequency is the amount of attestation, as discussed below. But stability, the shared correlate of frequency and word length, can be estimated directly from our data. Stability, in the specific sense used here, is defined in Materials and Methods. Results are given in Table 3 (and repeated in S8 File). Unlike word length, which requires no more than one language per genus, stability requires at least two languages per genus for its calculation, so for stability we generally had fewer genera available, the number ranging from 78 (for the item ‘round’) to 376 (for the item ‘fire’).

Just as for mean word length across items, the ranks of stability in the two hemispheres can be correlated in order to gauge the degree to which stability can be claimed to be inherent to the items. The result is a Spearman’s rank correlation coefficient of ρ = 0.73, similar to the value ρ = 0.76 for mean word length.

The large ASJP database mainly consists of a 40-item subset of the 100-item Swadesh list originally selected based on a different estimate of stability and a much smaller initial dataset of just 245 languages [3]. The similarity measure feeding into this previous stability estimate was binary (similar vs. not similar). Items were judged to be similar if at least two consecutive consonants (contiguous or interrupted by any vowel symbol(s)) in the respective words were identical. For items having only two or three symbols, vowel symbols were also taken into account. This earlier similarity criterion would in most cases identify cognates and thus focus on retention vs. replacement of whole items, whereas the LDND-based similarity measure is sensitive to both phonological changes, incurring differences between some symbols, and lexical changes, typically causing most, if not all, symbols to become different.

Our earlier stability estimates are mainly of historical interest, but since they had direct consequences for the selection of data going into the ASJP database it is of some interest to compare the earlier to the current estimates. The two correlate well (ρ = 0.82) and, more interestingly, a selection of the 40 most stable items using the current estimates would only be different with regard to 6 members. Thus, an updated list would include ‘bird’, ‘eat’, ‘head’, ‘moon’, ‘mouth’, and ‘what’ and not include ‘come’, ‘full’, ‘mountain’, ‘person’, ‘see’, and ‘star’.

Stability (Table 3) and mean word length (Table 2) are negatively correlated (ρ = -0.50). Since shorter words are expected to more frequent, and more frequent words are expected to be more stable, the correlation may be at least partly mediated by frequency. The correlation is not an artifact of the definitions, since the distance measure (LDND) on which stability is based controls for word length.

### Synonyms

A common feature of lexical change is the competition of word forms over the same semantic space [48, 49]. If a new word form is successful in replacing an older one, its proportion of the total frequency of the two forms is expected to rise over time in the shape of an S-curve [49], eventually taking over completely. A lexicographer recording a language during the period of change is likely to record the competing forms as being synonymous. Thus, a possible fingerprint of instability of the forms expressing a given concept is the degree to which the concept exhibits synonymy across languages. We would expect, then, that the amount of synonymy is correlated with stability. Moreover, if synonymy is a good proxy for stability it should further be negatively correlated with word length, given that word length is related to stability via frequency. In order to test these hypotheses, we measured synonymy as the percentage of doculects pertaining to a given ISO-639-3 language exhibiting two or more synonyms, and included this information in the online data posted at https://doi.org/10.5281/zenodo.6344023. We ignored the presence in the data of a third or fourth etc. synonym since high similarity in meaning is unlikely to hold for more than two words—an excessive recording of several words for the same meaning is thus more likely to reflect a characteristic of the lexicographer’s practice rather than a fact about the language.

As with mean word length and stability, synonymy for each item is averaged across languages within genera and then across genera. The rank correlation between the Eastern and Western Hemispheres is a substantial ρ = 0.62, showing that synonymy is a reliable characteristic of meanings.

The correlation between synonymy and stability is ρ = -0.41. The sign is as expected since more cases of synonyms across languages would imply less stability. It is prudent, however, to pause and consider the fact that stability and synonymy as defined here are not independent measures. It is part of the definition of synonyms that they differ in shape, i.e. they represent different word forms (but with a shared meaning). When we measure similarity between a word form in a given language A and each of two synonyms in language B and subsequently average the similarities to arrive at the similarity in the forms for a concept in A and B, then this similarity is by necessity never 1 (identity)—this would require the two synonyms in B to also be identical to each other, in addition to being identical to the form in A. So synonymy incurs dissimilarity and thus contributes directly to measured instability. This factor was controlled by recalculating stability using only the first synonym of each word and ignoring the second synonym (if any). The correlation between the two versions of stability is above 0.99, and the correlation between synonymy and the one-synonym version of stability is only slightly lowered, to ρ = -0.39. The negative correlation between synonymy and stability is therefore an empirical property of meanings and not a definitional artifact. It also replicates the negative correlation reported in Ref. [51]. However, synonymy correlates a negligible ρ = -0.09 with mean word length.

### Attestation

The last relevant property of items observable in the ASJP database is the percentage of languages in which a given item is attested. As usual, the percentage is averaged across languages within genera and then averaged across genera. Attestation depends to some extent on frequency because infrequent items are unlikely to be encountered and recorded in sources not relying on direct elicitation. Attestation also depends on the practices of researchers, who may be following not only their own judgments but also conventions that differ between language groups and geographical areas. For instance, the Concepticon compilation of concept lists used for lexical elicitation [50] includes more than 100 lists that were designed for specific areas. Attestation can be formalized as a special case of frequency in which the corpus consists of all the items used by the researcher, and the estimated frequency of an item takes the value 1 if the given item occurs in this corpus, and the value 0 otherwise. Despite its radical simplification as a measure of frequency, attestation correlates a respectable ρ = 0.55 between the Eastern and Western Hemispheres, suggesting that attestation reflects reliable properties of meanings to almost the same extent as mean word length, stability, and synonymy. Attestation correlates ρ = -0.37 with mean word length, ρ = 0.67 with stability, and ρ = -0.36 with synonymy. The correlation with stability is the strongest of the six correlations among the four variables observed here, and replicates the positive correlations between frequency and stability found in previous studies [46, 47, 51]. The negative correlation with mean word length is consistent with Zipf’s law and also with more recent findings [52, 53]. The negative correlation with synonymy is opposite from the positive correlation between frequency and synonymy found in Ref. [51], but these results may not be comparable since ‘synonyms’ in the latter study is a cover term that also includes hyponyms, hypernyms, and metaphorical synonyms, and the data come from dictionaries of 5 Germanic languages with long literary traditions.

### Semantics-syntax

While nouns, verbs, adjectives, and function words in the English encoding of the Swadesh list do not necessarily correspond to similar formal categories in other languages and are therefore better characterized semantically as concepts referring to things (in a broad sense), actions, property concepts, and functions (or ‘other’), we refer to them using parts-of-speech labels for convenience here. The 100 Swadesh items include 54 nouns, 19 verbs, 15 adjectives, and 12 function words (‘I’, ‘you’, ‘we’, ‘this’, ‘that’, ‘who’, ‘what’, ‘not’, ‘all’, ‘many’, ‘one’, ‘two’) as delimited by [51]. Table 4 addresses whether items in the four categories differ in mean word length, stability, synonymy, and attestation.

**Table 4:** Mean word length, stability, synonymy, and attestation by part of speech.

Variable	Chisq(3)	Nouns	Verbs	Adjectives	Function
Mean word length	27.76	4.50	4.44	5.09	3.92
Stability	18.68	25.00	20.38	17.83	23.59
Synonymy	45.70	10.53	15.65	15.13	19.03
Attestation	15.56	86.50	80.07	78.00	79.37
N		54	19	15	12

The first column gives the chi-square values (with 3 degrees of freedom) yielded by Kruskal-Wallis tests of differences between categories. Differences are significant (p < .01) for each variable. The remaining columns give the mean value of each variable averaged across items in each category. Nouns have the highest stability and attestation, the fewest synonyms, and moderate length. Verbs are moderate on all four variables. Adjectives are the longest, the least stable, and the least attested, with a moderate proportion of synonyms. Function words are the shortest and have the most synonyms, with relatively high stability and moderate attestation. The greater stability of nouns and function words than verbs and adjectives is consistent with differences among parts of speech found in earlier studies [46, 47, 51].

### Morphology

The ASJP word lists do not show morphological segmentation of words. Transcribers were instructed to remove inflectional morphology, and hyphens or functionally equivalent symbols that might be used to indicate morpheme boundaries are not part of the word lists. Thus, the data is not intended for analyses of a morphological nature, and any such analyses must be approached in indirect ways and considered preliminary. Still, we venture to ask whether systematic, cross-linguistic morphological patterns might account for the variation in mean word length across languages and between items reported in previous sections.

The 40 meanings on the list used here for computing a mean word length generally represent simple concepts that are usually not expected to be encoded through morphologically complex forms. Still, it is an open question whether morphological material like derivation, compounding or residual inflectional morphology nevertheless has an impact on our results for cross-linguistic variation in mean word length. We have carried out three experiments to investigate this issue, and all suggest that we would not get qualitatively different results even if our data were encoded in such a way that we could control for morphology and directly measure the length of lexical morphemes. All data pertinent for replication of the experiments, when not provided elsewhere, are in S10 File.

Experiment A1. If morphology is important for differences in mean word length, we might expect that the number of different available word formation strategies (WFSs) in a language correlates with mean word length (MWL). The worldwide morphological survey of Ref. [54] allows for a correlation of these two variables for a sample of 51 languages. The result indicates the absence of any significant relation (*r* = -0.1045, *p* = 0.4654). This study included data on 20 different WFSs. A later study [55] includes as many as 100 WFSs and more languages, 73 of which have MWL data in our dataset, but this sample is restricted to Europe. For this data, a correlation of the number of WFSs (variously called ‘structural richness’ and ‘saturation’ by the authors) and MWL yields *r* = 0.2392. An apparently (barely) significant *p* = 0.0415 is deceptive because of strong geographical and genealogical dependence within the sample, so this result goes in a similar direction, indicating that the importance of morphology is at most marginal.

Experiment A2. The procedure of Experiment A1 may be criticized for being naive in the assumption that a raw number of WFSs meaningfully summarizes the potential impact of morphology. An alternative assumption is that it is the presence of particular WFSs which (potentially) matters. In order to shift the attention to individual WFSs we used the worldwide dataset [54] to run a t-test for each particular WFS on the difference in mean MWL for the sets of languages possessing or not possessing the given WFS. The results (detailed in S10 File) show that only one of the 20 WFSs produced a t with p < 0.05, which is expected on average under the null hypothesis.

Experiment A3. For the purposes of this experiment we assume that the five cross-linguistically shortest items on the 40-item list (‘I’, ‘you’, ‘water’, ‘come’, and ‘tree’, according to Table 2) will at most sporadically carry morphological material. If an analysis using only these items for calculating MWL replicates an earlier analysis using all 40 items, it would then suggest that morphology had limited importance in the earlier analysis. We replicated results reported in Table 1 above, carrying out correlations between mean word length, log inventory, and log population across macroareas and languages using only the five shortest items for MWL calculations. A detailed comparison with the previous results is provided in S10 File. Crucially, the correlations are qualitatively similar, even if weaker, and the one significant correlation across macroareas, namely the one between word length and log population (earlier *r* = -0.92, now *r* = -0.84), remained significant, even if the level shifted (from *p* < .01 to *p* < .05).

Taken together, the three experiments indicate that we can meaningfully make generalizations about cross-linguistic variation in mean word length based on the ASJP word lists without somehow controlling for morphology. Morphology, when present, obviously contributes to the measured length of a word, but the presence or absence of particular morphological tendencies does not seem to be a major determinant of cross-linguistic variation in word length.

Moving on to variation across items (rather than across languages), we note, looking at Table 2, that part of speech membership seems to matter for the word length of items relative to one another. The pronouns ‘I’ and ‘you’ are the shortest, with ‘we’ also in the shorter end. Other function words also tend to be short. Adjectives, on the other hand, tend to be long. In the middle range we find the numerals ‘one’ and ‘two’ close to one another in rank. Morphology may be an explanatory factor here. Perhaps function words tend not to carry morphology whereas adjectives do, and perhaps the numerals ‘one’ and ‘two’, when they carry morphology, tend to carry similar morphological material. Such tendencies would contribute to the patterns we observe. Comparative research on the morphological treatment of different types of lexical items across languages is not available, so confirmation must be left to the future, but at least we have a testable hypothesis.

### Stability of word length

Since the table of mean word lengths in the WALS-style data sheet (https://doi.org/10.5281/zenodo.6344023) is formatted like a feature in WALS itself, the stability of word length can be assessed by the same method previously applied to WALS features [19]. The result is a stability of 25.0, which is below the median stability of WALS features but above the 25th percentile.

This result carries a methodological limitation, however. The data table partitions the continuous variable of word length into eight discrete categories, but the number of categories can influence estimated stability. The formula for stability is S = (R–U)/(1 –U), where S is the stability of a given feature, R is the proportion of pairs of related languages (in the same WALS genus) that match on the given feature, and U is the proportion of pairs of unrelated languages (in different WALS families) that match on the given feature. As the number of categories becomes very large, the quantities R and U both approach 0 and therefore so does stability. In WALS itself, the number of categories ranges from two to nine, with a mean of 4.66. For word length, when the number of categories is reduced from eight to five by deleting the cutoffs at 3.5, 4.5, and 5.5, stability of mean word length increases to 35.0, slightly above the median. Moreover, the correlation across WALS features between stability and category number is only ρ = 0.-0.13, indicating that real differences among features in stability are large enough to offset the methodological effect of category number. It seems safe to conclude that mean word length is a middle-of-the-road feature in terms of stability.

## Outstanding questions

The questions addressed in this paper were the ones that we could reasonably address through a dataset consisting in word lists for 5300+ languages of which 1200+ cover up to 100 items and the rest cover up to 40 items. While the per-language information is highly restricted, the coverage of languages is unparalleled in related studies.

The nature of the data is best suited to study *differences between languages*. We were able to discern worldwide areal patterns while at the same time seeing phylogenetic signals in word length data. While both effects are clearly there, it would be desirable to be able to quantify the respective effects of areality and inheritance on word length. Currently we do not have a good solution to this difficult problem, but a possibility may be the modeling of the evolution of word length along the branches of phylogenies, looking to see how often a neutral model of evolution would account for the changes as opposed to selection pressure from neighboring languages. Since the location of neighboring languages at different time periods would also have to be reconstructed, which could impose an insurmountable challenge, we may be forced to carry out the investigation *in silico*, through simulations where the probability parameters leading to realistic distributions of languages and their properties are investigated, cf. [20, 56]. With respect to the hypothesis that having very long words is generally a recessive feature, whereas having very short words seems to be innovative when it occurs, it would again be useful to take a phylogenetic perspective.

The inverse relation between word length and population, which holds between macroareas but not within macroareas or families, contradicts the uniformitarian assumption that currently observable processes can be extrapolated to explain patterns from the distant past. This contradiction undermines uniformitarian theories like [33]. It also raises the question of what historical event or process could generate a correlation strong enough to persist today at the level of differences between macroareas, while producing no correlation within macroareas or families. One possibility is the transition to agriculture in different parts of the world, initially in the Fertile Crescent of Southwest Asia, and then in Northeast China, Highland New Guinea, tropical regions of the Americas, and the Eastern Woodlands of the USA [57]. We speculate that word length was highly affected by initial population growth as prehistorical peoples became sedentary food producers but was less affected by subsequent population growth. The effect of population must approach an asymptote, because populations of some languages have continued to increase after the Neolithic with no visible effect on word length within groups. As described above for a different question, computational modeling may be an aid in assessing the viability of such a scenario.

Correlations between word length and sizes of phonological segment inventories showed stronger support for an inverse relation than hitherto available. Nevertheless, a deeper relation may exist between word length and information load as measured in bits per phoneme as suggested in Ref. [58]. This paper drew upon a lexical resource limited to Eurasia, pressed by a greater need for data on individual languages. Since no attempts are made to control for non-independence in the data, any conclusion based on those values should be taken with a grain of salt. Nevertheless, a measure taking into account the distribution of segments over words could have a potentially greater explanatory value than a mere count of segments when looking a variation in word length.

As regards *differences between items* the limitations imposed by working with short word lists were more strongly felt. We were able to establish that there are differences in word length and stability inherent to different meanings and sematic-syntactic categories, and also that most of the correlations expected from frequency can be extended to amount of attestation. The attestation measure has the advantage of being available for 5289 languages in the present database, while most previous data and theory on the relation between frequency and word length is ultimately based on only 26 languages [46, 47, 51, 52, 59]. The available corpora are not sufficient to investigate the degree to which the observed effects of attestation are driven specifically by differences in frequency. We are in an even worse position to follow up on the suggestion [60] that information is actually a better predictor of word length than frequency. In order to revisit the role of frequency (or information) we would need enough corpora to get a decent coverage of linguistic diversity, preferably corpora resembling naturalistic speech about everyday events. While corpora containing religious, scientific or political texts exist in many languages (such as Bible translations, Wikipedia, and the Universal Declaration of Human Rights), they are problematical as sources for word frequency data. Movie and TV subtitles are a more appropriate source, and some of the way for the use of this kind of resource has been paved [61]. Cross-linguistic morphological tendencies specific to different parts of speech may contribute to differences in word length between items, but much more comparative morphological research is needed before this hypothesis can be confirmed, and this research will face many challenges, including difficulties in defining the concept of ‘word’ cross-linguistically [62].

## Conclusions

Since the previous section outlined major outstanding issues of the understanding of word length variation within and across languages we can limit this conclusion to actual findings.

Word length exhibits the normal behavior of a typological feature of language, with a sensitivity to areal influence which is detectable over a ~5000 km range, but the areal signal is to some degree counterbalanced by the maintenance of a strong genealogical signal and a measurable stability which places it close to average among other typological features. The more extreme cases of long words interestingly align with Nichols’ Pacific Rim area [28], and having long words seems to be a recessive feature. Word length is significantly (p < 0.01) inversely correlated with (log) population size, a finding which resonates with other observations suggesting an inverse relation between language complexity in general and population size [63]. It appears that population growth, mainly in areas that were also affected by early shifts to a farming subsistence such as South and Southeast Asia, Africa, and West and Southwest Eurasia, has been driving words to become shorter in many of the world’s languages.

Some meanings tend to be represented by words that are shorter than for some other meanings, with the range represented by the extreme cases of the pronoun ‘I’, having an average length of close to three segments, and the meaning ‘round’, with an average length of close to six segments. These tendencies are robust across world areas, suggesting that there is something universally inherent to these meanings which is shared across cultures. A similar kind of statistical universality holds for stabilities of items, i.e. the degree to which words for different meanings tend to change shape in the course of evolution of languages. The most stable items on the Swadesh list include ‘I’, ‘water’, ‘you’, ‘louse’, and ‘eye’, while the least stable include ‘many’, ‘lie’, ‘small’, ‘that’, and ‘bark’. When grouping items into semantic-syntactic categories we find that words referring to things (in a broad sense) occupy the higher end of the stability scale, contrasting with words referring to property concepts, which are on the lower end. The latter, on the other hand, tend to be long, and in this regard are in contrast with function words, which tend to be short. Less stable meanings have a noticeable tendency to be represented by synonyms in word lists, which is expected given that synonymy is a signature of competition between word forms over a semantic space. The degree to which frequency as conventionally defined drives word length and stability is a question we could not address directly with the data used for this study, but we found that the average percentage of attestation of different items qualifies as a simplified measure of frequency, showing a positive relation with stability and a negative relation with word length.

In addition to these observations on the conditions for word length we offer a dataset of 5289 languages along with a subset of the data accommodated to the WALS framework for use in future typological studies of word length, at https://doi.org/10.5281/zenodo.6344023.

## Supporting information

S1 File Data preparation. (PDF)

S2 File On distributions of phoneme inventory sizes. (PDF)

S3 File On stability measures. (PDF)

S4 File Plot using WALS family definitions. (PDF)

S5 File Maps showing mean word length averaged over families within Autotyp areas. (PDF)

S6 File Correlations within families and macroareas. (PDF)

S7 File Differences between macroareas. (PDF)

S8 File Summary data on mean word length, stability, synonymy, and percent attestation. (PDF)

S9 File Why properties of items are estimated by averaging over WALS genera. (PDF)

S10 File Morphology. (PDF)
