# Supplementary material for: Cross-linguistic conditions on word length
Source: PLoS One. 2023 Jan 27;18(1):e0281041. doi: 10.1371/journal.pone.0281041 (PMC9882889; doi:10.1371/journal.pone.0281041)
Supplement: S1 File — (PDF) [file pone.0281041.s001.pdf]

S1: Data preparation

The ASJP data raw table. The basic materials drawn upon are provided as a file called Data-01 ASJP data raw.txt. This contains a row per ISO 639-3 language, with columns containing the following information (column names, in italics, precede the description of each column).

- *iso*: the ISO 639-3 code;
- *names*: ASJP names of doculects pertaining to the ISO-code, separated by commas;
- *wals_code*: code from WALS [1];
- *wals_fam*: WALS family classification (for languages not in WALS this is the family which the editors of the ASJP database assume that a language would belong to in the WALS classification scheme, which may not necessarily agree with the decision that the editors of WALS themselves would eventually take);
- *glot_fam*: Glottolog family classification [2];
- *wals_genus*: WALS genus classification (the hedges pertaining to the wals_fam column also apply here);
- *lat*: geographical latitude, using as a proxy the latitude of the first occurring ISO-639-3 representative in the order of languages in ASJP;
- *lon*: geographical longitude of the first occurring ISO-639-3 representative;
- *pop*: population size of the first occurring ISO-639-3 representative—this is indicated in the ASJP database whenever it is known and a language is still spoken (otherwise NA);
- *forty_hundred*: abbreviations H or F signaling whether a wordlist was filled for the 100 items of the Swadesh list or just the 40 items of the ASJP selection (H is used if at least one of the lists aggregated over is a 100-item list);
- *I*, *you*, *we*, etc.: mean word length for each of the 100 items in the Swadesh list with minimal aggregate units in each cell and NA for missing values;
- *syn_I*, *syn_you*, *syn_we*, etc.: the percentage of doculects with two or more synonyms;
- *perc_att_40*: a percentage for the total number of attestations among the 40 items;
- *perc_att_100*: a percentage for the total number of attestations among the 100 items;
- *forty_mean*: word length averaged over the 40 ASJP items;
- *hundred_mean*: word length averaged over the 100 items on the Swadesh list, but NA if the value in the forty_hundred cell is F;
- *area*: assignment of a language to one of the 24 world areas as defined in Autotyp [3];
- *continent*: assignment of a language to one of the 10 'continents' (macroareas) of Autotyp;
- *macrocontinent*: assignment of each language to one of the 4 'macrocontinents' (super-macroareas) of Autotyp.[1]

[1] We used the Autotyp metadata found on GitHub (accessed 2021-09-22). The area definitions of Autotyp are provided at https://github.com/autotyp/autotyp-data/blob/master/metadata/Register.yaml. If a language in ASJP is also found in Autotyp it inherits its areas assignment directly from Autotyp (except a handful of cases where wrong ISO 639-3 codes are used in Autotyp or where the geographical information is erratic—in such cases a language was treated as not being present in Autotyp). If a language in ASJP is not found in Autotyp (or has been removed as one of the problematical cases) it inherits its area assignments from the geographically closest language in Autotyp. The geographical metadata of Autotyp is at https://github.com/autotyp/autotyp-data/blob/master/data/Register.csv.

Even if not all doculects are used in our analyses, we include them all in this table, except (1) doculects that have "PROTO" as part of their ASJP name, (2) are tagged as ARTIFICIAL, FAKE or SPEECH_REGISTER in their WALS genus classification, (3) lack a Glottolog classification, (4) are tagged as Mixed Language, Spurious, Unclassifiable or Artificial Language in their Glottolog family classification or (5) are only documented in the year 1700 or earlier. We do not select the languages in the table based on the number of items attested since such a selection can be made using information on the percent attested items already in the table. The results reported in this paper are based on languages with at least 20 of the 40 ASJP items attested, a cutoff chosen for the reasons described in the section 'Criterion for data inclusion' below. The results reported also exclude languages classified by WALS as Pidgins & Creoles.

The data table is produced using a script called ASJP_data_raw.R which, along with associated files, is available at https://github.com/Sokiwi/WordLength.

The WALS data table. We also provide a file called Data-02 WALS data.txt. This is intended as a table that can be directly appended to WALS. So it only includes a WALS code, a continuous word length value and a corresponding categorical value for each of the languages already in WALS. The values are derived from the 40 items only, although they can come from both 40-item and 100-item lists. They are based on averages over the minimal aggregate units of analysis. In order to generate categorical values from the continuous data we applied evenly spaced cutoff points from 3 to 6 segments, in steps of 0.5. Table S01-1 shows the categorical values, their definitions, and their distribution over languages.

Table S01-1. Values, definitions, and distributions pertaining to the WALS data table

category label (value)	mean word length (MWL)	number of languages
extremely short	MWL < 3	120
very short	3 ≤ MWL < 3.5	363
Short	3.5 ≤ MWL < 4	644
moderately short	4 ≤ MWL < 4.5	614
moderately long	4.5 ≤ MWL < 5	320
Long	5 ≤ MWL < 5.5	155
very long	5.5 ≤ MWL < 6	66
extremely long	6 ≤ MWL	27

In principle, any language carrying a WALS code is potentially included in the WALS data table. In actual fact, a language (as defined by its WALS code) ends up getting included in this data table if and only if at least 20 out of the 40 ASJP items are attested for the language. We arrived at this criterion by striking a balance between minimizing error in the mean word length estimate and maximizing the number of languages included, as described in the next section.

The data table is produced using a script called WALS_data.R which, along with associated files, is available at https://github.com/Sokiwi/WordLength.

Criterion for data inclusion. How complete should a word list be in order for a mean word length estimate based on that list to be included in the data for this paper? Naturally one would like to be able to include as many data points as possible, but one would also want to keep the error due to missing data at a minimum. In order to estimate the growth of error as a function of

missing items we first selected the 647 WALS languages for which all 40 items are attested. For each possible number of missing items (denoted M, where $1 \leq M \leq 39$) we sampled 10,000 languages (with replacement), excluding M random items from each, calculated mean word length, and found the absolute difference between the mean word length of the sample and that of the full 40 items. We then took the mean of these differences and plotted these as a function of M. The result is shown in the left-hand panel of Figure S01-1.

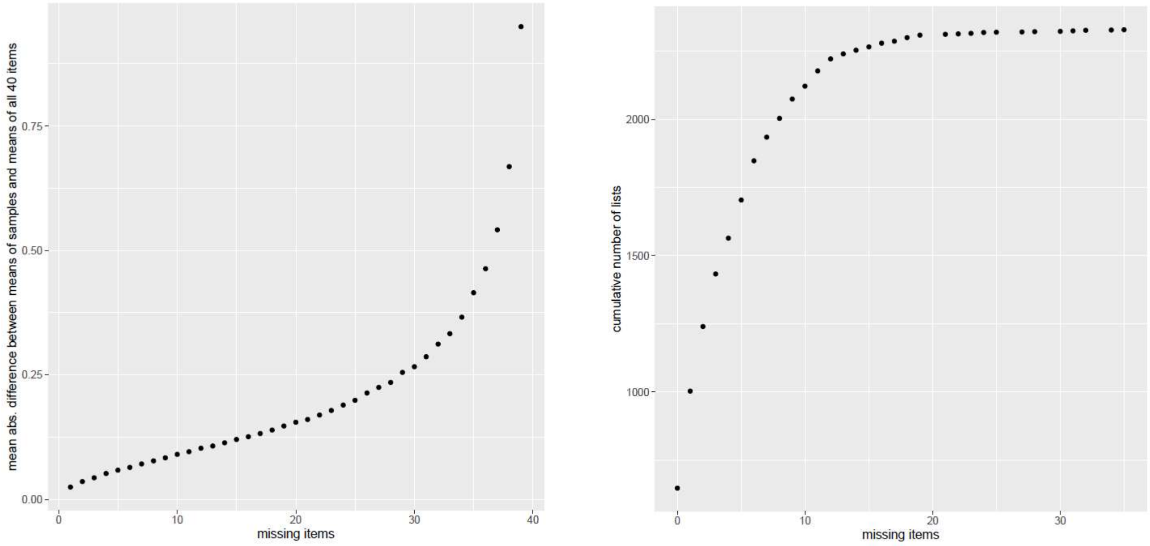

Fig. S01-1. Left: Mean difference between means of samples and mean of 40 items as a function of the number of missing items. Right: Cumulative number of lists as a function of missing items.

The left-hand panel of Figure S01-1 indicates that the error begins to accelerate somewhere in the vicinity of 25 missing items. The right-hand panel shows the cumulative number of WALS languages as a function of missing items and demonstrates that the returns radically diminish with 20 or more missing items. Since this is a value at which the error curve still has not begun to accelerate it seems like an appropriate cutoff for the number of missing items allowed if a language is to be included. Applying this cutoff we excluded 20 languages.

References

1. Dryer, MS, Haspelmath M, editors. The world atlas of language structures online. Leipzig: Max Planck Institute for Evolutionary Anthropology; 2013. Available from: http://wals.info.

2. Hammarström H, Forkel R, Haspelmath M, Bank S. Glottolog 4.4. Leipzig: Max Planck Institute for Evolutionary Anthropology; 2021. [cited 2018 Jan 18]. Available from: https://doi.org/10.5281/zenodo.4761960.

3. Bickel B, Nichols J, Zakharko T, Witzlack-Makarevich A, Hildebrandt K, Rießler M et al. The AUTOTYP typological databases. Version 0.1.0; 2017. Available from: https://zenodo.org/record/3667562#.YineCJYo9EY doi: 10.5281/zenodo.3667562
