# Supplementary material for: Cross-linguistic conditions on word length
Source: PLoS One. 2023 Jan 27;18(1):e0281041. doi: 10.1371/journal.pone.0281041 (PMC9882889; doi:10.1371/journal.pone.0281041)
Supplement: S2 File — (PDF) [file pone.0281041.s002.pdf]

S02: On distributions of phoneme inventory sizes

The variable representing phoneme inventory sizes is log-transformed (base 10) and scaled by subtracting the mean and dividing by the standard deviation. Figure S02-1 shows the logged and scaled distribution in comparison with the original distribution.

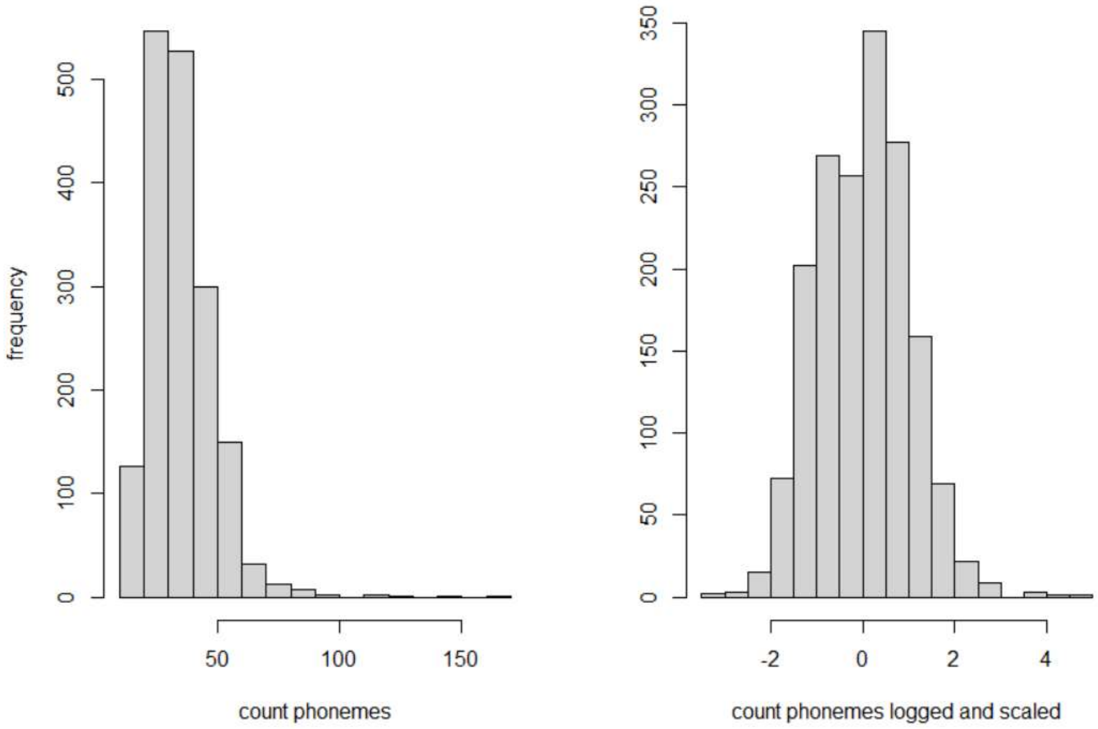

Fig. S02-1. Comparison of the distributions of original and log-transformed values for phoneme inventory sizes
