# Supplementary material for: Cross-linguistic conditions on word length
Source: PLoS One. 2023 Jan 27;18(1):e0281041. doi: 10.1371/journal.pone.0281041 (PMC9882889; doi:10.1371/journal.pone.0281041)
Supplement: S3 File — (PDF) [file pone.0281041.s003.pdf]

S03: On stability measures

Here we show how the definition of stability for items in this paper is related to the stability measure used in Ref. [1] for discrete features in WALS. Both measures are based on pairs of languages. For features, the stability formula is

(1) $S = (R - U)/(1 - U)$,

where S is stability, R is the proportion of related pairs (in the same WALS genus) that match on a given feature, and U is the proportion of unrelated pairs (in different WALS families) that match on the given feature. This equation can be restated in terms of differences rather than matches by letting $R' = 1 - R$ and $U' = 1 - U$; substitution into (1) and rearrangement lead to

(2) $S = 1 - R'/U'$.

For items, stability is the similarity 1 – LDND between languages in the same genus. The numerator in LDND is LDN, which is analogous to R′ because it is a distance between related languages. The denominator in LDND is an average distance between unrelated meanings, which is analogous to U′, although the analogy is not as close as in the numerator. Consequently, the definition of stability for items as 1 – LDND is analogous to (2).

References

1. Wichmann S, Holman EW. Temporal stability of linguistic typological features. München: LINCOM Europa; 2009.
