# Supplementary material for: Cross-linguistic conditions on word length
Source: PLoS One. 2023 Jan 27;18(1):e0281041. doi: 10.1371/journal.pone.0281041 (PMC9882889; doi:10.1371/journal.pone.0281041)
Supplement: S4 File — (PDF) [file pone.0281041.s004.pdf]

S04: Plot using WALS family definitions

Fig. S04-1 demonstrates that Figure 1 would not look appreciably different using family definitions from WALS rather than from Glottolog. Since WALS is more inclusive than Glottolog in a few cases, the first bin for related languages shows a slightly greater difference in word length when using the WALS classification. Other differences are so small as not to show up visually. Since Figure 1 already shows the effects of different distance measures we only plot distances as the crow flies for the present purpose of comparing effects of different classifications.

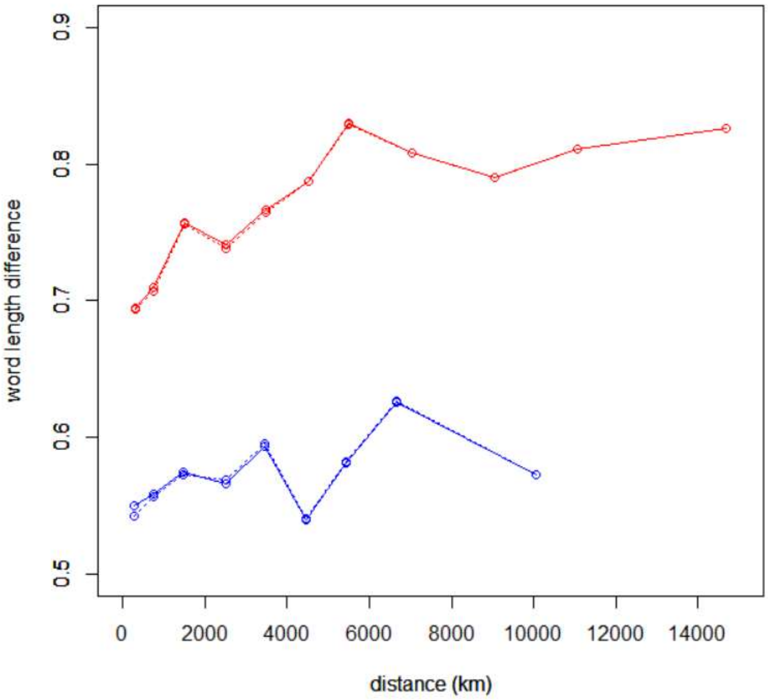

Fig. S04-1. Word length difference as a function of Great Circle Distance for unrelated (red curves) and unrelated (blue curves) languages, using WALS (solid curves) and Glottolog (dotted curves) family definitions. For both variables the means within bins are plotted.
