# Supplementary material for: Cross-linguistic conditions on word length
Source: PLoS One. 2023 Jan 27;18(1):e0281041. doi: 10.1371/journal.pone.0281041 (PMC9882889; doi:10.1371/journal.pone.0281041)
Supplement: S5 File — (PDF) [file pone.0281041.s005.pdf]

S05: Maps showing mean word length averaged over families within Autotyp areas

Each map shows values through the following color-coding: blue – 'short', light blue – 'moderately short', light red – 'moderately long', red – 'long'. The choice of locations for dots is based on the same minimum distance approach as used in the plot for WALS genera in Figure 2, but now without any implications of homelands—the choice merely signals where the main concentrations of the languages are. Each area is represented by the mean of mean word length of families in the area. If a family reaches into more than one area, the mean word length of those languages of the family that belong to a given area contribute their value to the overall mean word length of that area.

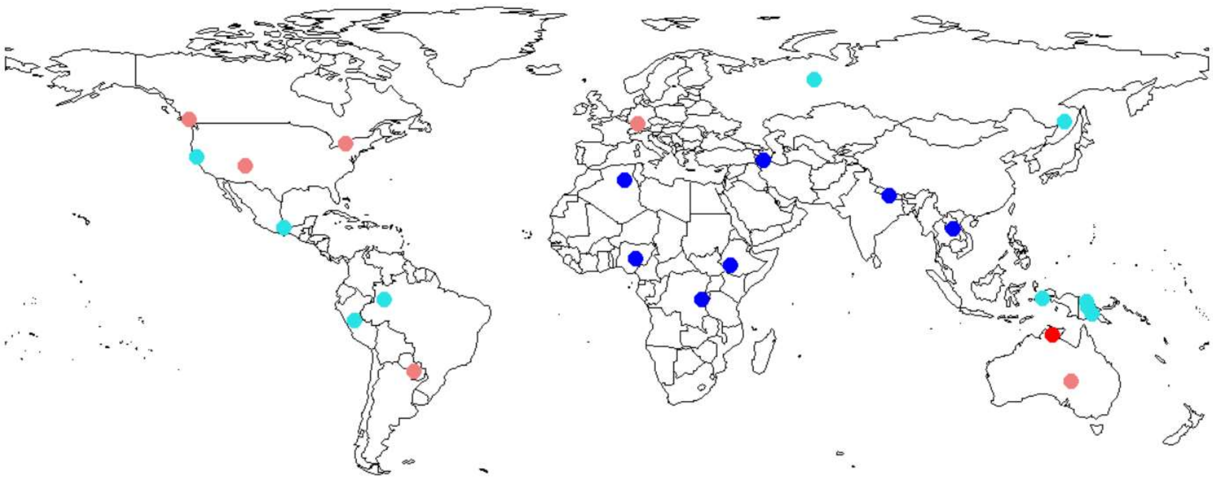

Fig. S05-1. Areas of the Autotyp 'area' category. From left to right: Alaska-Oregon, California, Basin and Plains, Mesoamerica, E North America, Andean, NE South America, SE South America, N Africa, African Savannah, Europe, S Africa, Greater Abyssinia, Greater Mesopotamia, Inner Asia, Indic, Southeast Asia, Oceania, N Australia, N Coast Asia, S Australia, N Coast New Guinea, Interior New Guinea, S New Guinea.

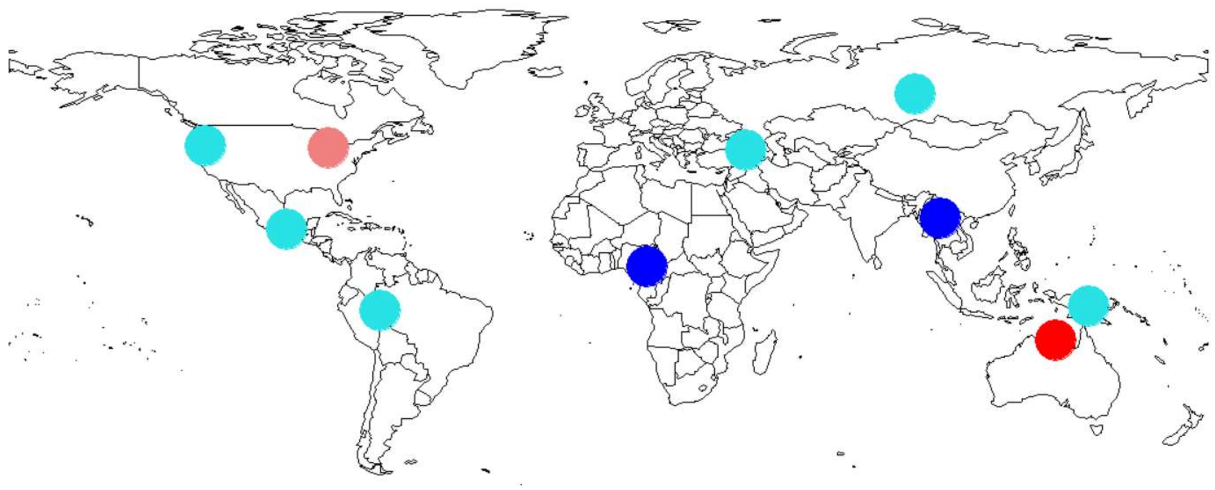

Fig. S05-2. Areas of the Autotyp 'continent' category. From left to right: W N America, C America, E N America, S America, Africa, W and SW Eurasia, N-C Asia, S/SE Asia, Australia, NG and Oceania.

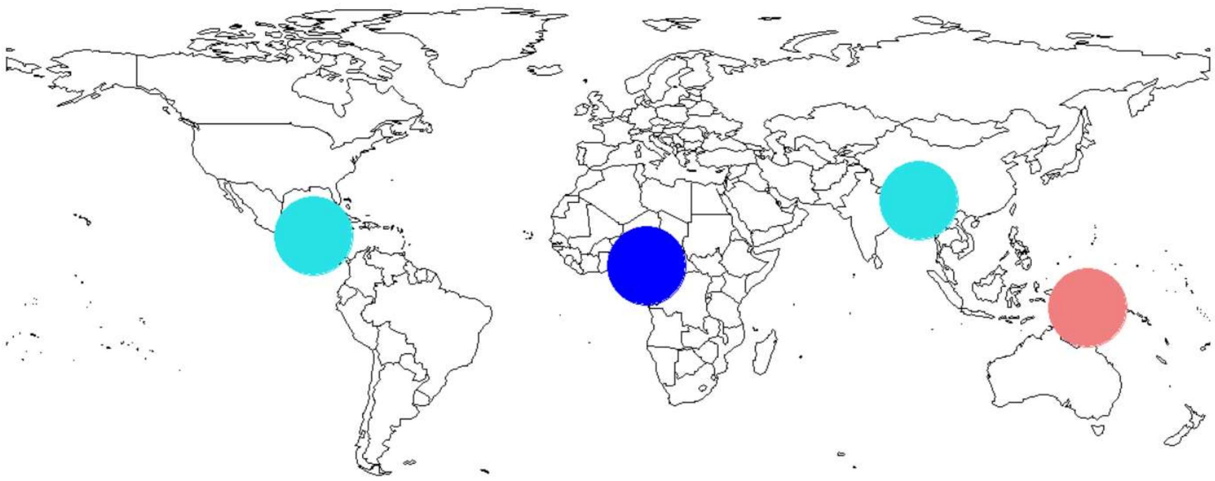

Fig. S05-3. Areas of the Autotyp 'macrocontinent' category. From left to right: Americas, Africa, Eurasia, Pacific.
