# Supplementary material for: Cross-linguistic conditions on word length
Source: PLoS One. 2023 Jan 27;18(1):e0281041. doi: 10.1371/journal.pone.0281041 (PMC9882889; doi:10.1371/journal.pone.0281041)
Supplement: S6 File — (PDF) [file pone.0281041.s006.pdf]

S06: Correlations within families and macroareas

Table S06-1 shows correlations between mean word length and log population within families with 20 or more members. There are 11 positive and 7 negative correlations, but (unrealistically assuming independence) only 5 are significant. The mean of the correlations is 0.0609.

Table S06-1. Correlations between mean word length and log population for larger families

Family	Correlation	p-value	N
Atlantic-Congo	0.234	< 0.0001	388
Indo-European	0.026	0.7829	113
Austronesian	0.291	0.0026	105
Afro-Asiatic	0.001	0.9940	98
Sino-Tibetan	-0.061	0.5982	78
Pama-Nyungan	0.131	0.3135	61
Tupian	0.065	0.6698	45
Mande	0.395	0.0106	41
Austroasiatic	-0.119	0.4771	38
Arawakan	0.007	0.9646	38
Nuclear Trans New Guinea	0.156	0.4179	29
Dravidian	0.469	0.0118	28
Nilotic	-0.139	0.5158	24
Uralic	-0.277	0.1906	24
Central Sudanic	-0.233	0.2962	22
Quechuan	-0.036	0.8768	21
Cariban	-0.298	0.1891	21
Turkic	0.484	0.0264	21

Table S06-2 shows correlations between mean word length and log population within macroareas. -There are three positive and three negative correlations, but (unrealistically assuming independence) only one is significant. The mean of the correlations is -0.0098.

Table S06-2. Correlations for mean word length and log population

Macroarea	Correlation	p-value	N
Africa	0.136	0.0005	656
Eurasia	-0.088	0.0553	475
S America	-0.024	0.6970	273
North America	-0.147	0.1147	117
Australia	0.038	0.6972	106
NG and Oceania	0.026	0.8218	79

Figure S06-1 shows scatterplots corresponding to the data in Table S06-2.

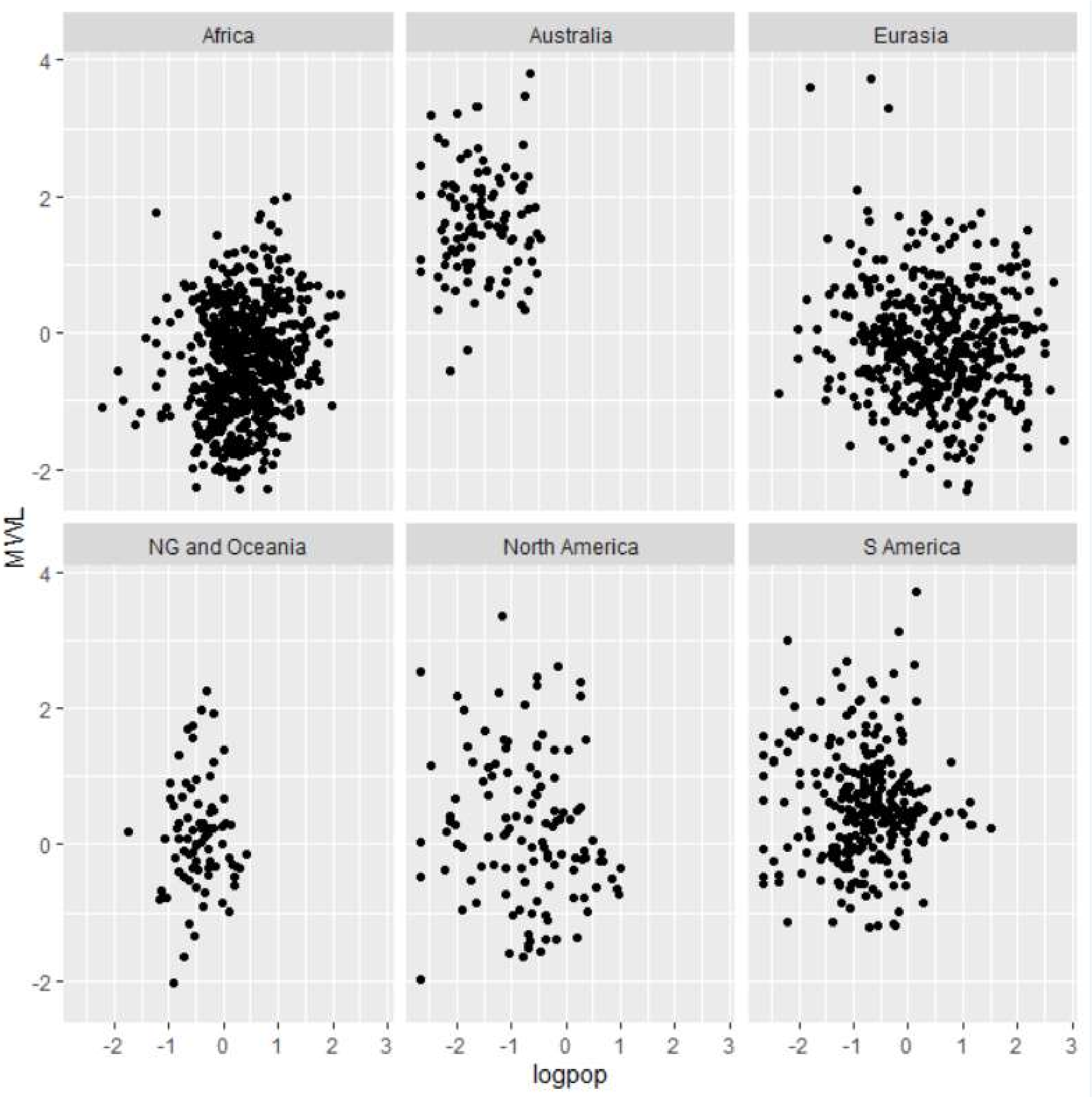

Fig. S06-1. Scatterplots of mean word length and log of population for different macroareas.

Below are results of a mixed effects regression model, using the lme4 R package [1], with mean scaled word length (forty_mean_s) as a function of scaled log population (log10pop_s) and scaled log inventory size (log10count_pho_s), with families nested into macroareas as random effect:

```
Linear mixed model fit by maximum likelihood  ['lmerMod']
Formula:
```

```
forty_mean_s ~ log10pop_s + log10count_pho_s + (1 |
macr_fact/fam_fact)
   Data: pho

     AIC      BIC   logLik deviance df.resid
  3615.5   3648.1  -1801.7   3603.5     1700

Scaled residuals:
    Min      1Q  Median      3Q     Max
-2.9092 -0.6093 -0.0288  0.5253  3.4817

Random effects:
 Groups             Name        Variance Std.Dev.
 fam_fact:macr_fact (Intercept) 0.4401   0.6634
 macr_fact          (Intercept) 0.5445   0.7379
 Residual                       0.4032   0.6350
Number of obs: 1706, groups:  fam_fact:macr_fact, 210; macr_fact, 6

Fixed effects:
                 Estimate Std. Error t value
(Intercept)       0.52606    0.30744   1.711
log10pop_s        0.08387    0.02359   3.555
log10count_pho_s -0.05231    0.02299  -2.276

Correlation of Fixed Effects:
            (Intr) lg10p_
log10pop_s  0.048
lg10cnt_ph_ 0.031  0.013
```

The t values above depend on the assumption that languages in the same family are mutually independent. A likelihood ratio test of the model with the inventory size effect against the model without the inventory size effect revealed a significant difference between models ($\chi^2(1) = 5.11$, $p < 0.05$). Similarly, a likelihood ratio test of the model with the population effect against the model without the population effect revealed a significant difference between models ($\chi^2(1) = 12.44$, $p < 0.001$). So in both cases there is sufficient evidence against the null hypothesis of model equivalence, assuming the independence of languages in the same family.

Comparisons of model fits, as measured by AIC and logLik, revealed that a removal of any of the variables relating to inventories, macroareas or families had adverse effects on the fit, which supports a model using all variables. Moreover, varying the slopes and not just the intercept, as in the model reported above, did not improve the fit either.

References

1. Bates D, Maechler M, Bolker B, Walker S. Fitting linear mixed-effects models using lme4. J Stat Softw. 2015;67(1):1–48. doi:10.18637/jss.v067.i01.
