# Supplementary material for: Cross-linguistic conditions on word length
Source: PLoS One. 2023 Jan 27;18(1):e0281041. doi: 10.1371/journal.pone.0281041 (PMC9882889; doi:10.1371/journal.pone.0281041)
Supplement: S7 File — (PDF) [file pone.0281041.s007.pdf]

S07: Differences between macroareas

We visualize the variation for each variable within macroareas through boxplots, cf. Figure S07-1 (prepared using the R package ggplot2 [1]). Africa, Australia, and Eurasia seem to be especially distinct with respect to the variables scaled mean word length (forty_mean_s) and scaled log inventory size (log10count_pho_s).

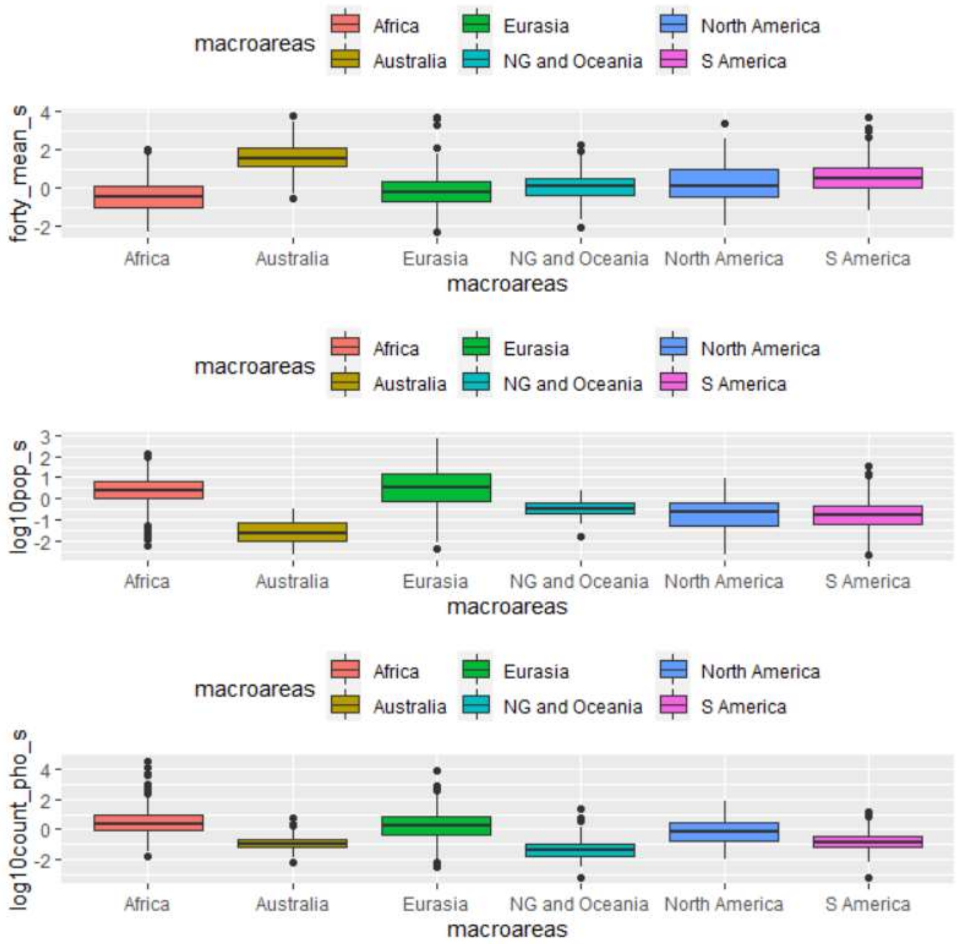

Fig. S07-1. Boxplots showing the distribution of values of our main variables with macroareas.

A one-way multivariate analysis of variance reveals whether, in general, there are some differences to be found. This analysis was carried out using the manova() function of R [2]. The following is the output of such an analysis.

```
                   Df  Pillai approx F num Df den Df    Pr(>F)
independent_var     5 0.73317   109.97     15   5100 < 2.2e-16 ***
Residuals        1700
---
Signif. codes:  0 '***' 0.001 '**' 0.01 '*' 0.05 '.' 0.1 ' ' 1
```

The *p*-value is practically zero, which means that if languages are independent within macroareas, we can safely reject the null hypothesis in the favor of the alternative one—at least one group mean vector differs from the rest.

References

1. Wickham H. ggplot2: Elegant graphics for data analysis. New York: Springer-Verlag; 2016.

2. Core Team. R: A language and environment for statistical computing. Vienna, Austria: R Foundation for Statistical Computing; 2021. Available from: https://www.R-project.org/.
