# Supplementary material for: Cross-linguistic conditions on word length
Source: PLoS One. 2023 Jan 27;18(1):e0281041. doi: 10.1371/journal.pone.0281041 (PMC9882889; doi:10.1371/journal.pone.0281041)
Supplement: S8 File — (PDF) [file pone.0281041.s008.pdf]

S08: Summary data on mean word length, stability, synonymy, and percent attestation

The word length data ('MWL') is also in Table 2 of the paper, and the stability data ('stab') is also in Table 3 of the paper.

Table S08-1. Summary data.

Item	POS	MWL	stab	%syn	%att
I	2	3.034	38.08	15.32	92.57
you	2	3.065	35.07	19.56	89.69
we	2	3.962	30.10	17.08	89.36
this	2	3.322	20.08	24.90	68.80
that	2	3.523	12.83	27.60	66.24
who	2	3.785	22.30	11.24	78.51
what	2	3.876	24.41	18.82	77.73
not	2	3.283	15.94	24.63	62.63
all	2	5.131	13.97	23.45	71.65
many	2	4.946	12.05	20.85	63.01
one	2	4.543	26.24	13.06	96.03
two	2	4.553	31.98	11.86	96.18
big	4	4.756	13.85	19.85	84.99
long	4	4.993	19.70	13.20	81.53
small	4	5.337	12.42	26.55	80.97
woman	1	4.620	20.16	15.74	91.12
man	1	4.765	22.52	15.53	88.94
person	1	4.446	23.40	14.27	79.41
fish	1	4.327	27.22	9.33	90.64
bird	1	4.568	24.53	8.45	94.78
dog	1	4.275	30.27	10.90	95.86
louse	1	4.143	34.56	9.65	83.67
tree	1	3.860	30.56	11.46	91.44
seed	1	4.393	14.52	14.02	66.76
leaf	1	4.474	28.06	12.67	89.92
root	1	4.825	19.76	10.16	87.60
bark	1	4.970	13.68	9.52	70.87
skin	1	4.452	25.95	13.03	91.03
flesh	1	4.289	17.93	9.37	75.85
blood	1	4.255	29.66	9.29	95.08
bone	1	4.268	31.43	8.24	92.18
grease	1	4.603	21.72	14.28	67.03
egg	1	4.418	21.34	7.60	92.23
horn	1	4.650	28.99	8.46	57.88
tail	1	4.489	20.90	6.70	85.87
feather	1	4.517	17.36	13.45	72.89
hair	1	4.563	18.28	13.77	87.51
head	1	4.317	26.70	11.37	96.28

ear	1	4.736	32.30	9.32	95.67
eye	1	4.141	34.26	10.43	97.96
nose	1	4.479	31.10	8.25	93.90
mouth	1	4.159	26.27	9.01	92.31
tooth	1	4.098	32.97	9.08	95.02
tongue	1	4.768	33.18	9.36	93.55
claw	1	5.302	18.99	9.62	57.16
foot	1	4.420	22.18	11.14	81.10
knee	1	5.501	26.02	10.4	82.69
hand	1	4.072	29.71	10.3	94.89
belly	1	4.357	15.22	12.97	85.86
neck	1	4.796	17.36	11.47	86.96
breast	1	4.195	27.22	12.02	88.31
heart	1	5.019	19.11	8.50	77.00
liver	1	4.596	28.23	7.13	80.35
drink	3	3.877	28.86	12.28	85.82
eat	3	3.841	24.45	18.41	94.10
bite	3	4.548	21.92	17.48	78.26
see	3	4.059	19.61	16.05	90.57
hear	3	4.648	25.77	12.44	84.86
know	3	4.783	16.21	11.58	73.87
sleep	3	4.568	23.94	10.70	89.75
die	3	4.187	29.12	16.32	85.06
kill	3	4.611	15.21	15.50	81.36
swim	3	5.094	19.60	12.87	70.02
fly	3	5.026	19.76	11.78	72.58
walk	3	4.513	18.06	17.73	74.75
come	3	3.737	22.04	16.39	89.27
lie	3	4.764	12.18	15.85	62.38
sit	3	4.775	15.50	13.84	82.40
stand	3	5.095	17.12	15.86	78.58
give	3	4.022	23.56	15.01	85.05
say	3	3.848	16.60	23.94	75.96
sun	1	4.402	27.53	11.27	93.15
moon	1	4.551	26.43	7.37	90.68
star	1	5.513	24.17	9.59	90.49
water	1	3.526	36.21	10.65	98.23
rain	1	4.158	25.58	6.55	92.34
stone	1	4.298	31.99	12.53	95.26
sand	1	4.977	17.32	8.89	86.38
earth	1	4.314	22.29	14.26	75.76
cloud	1	5.055	14.72	6.99	82.63
smoke	1	4.601	23.58	7.52	91.40
fire	1	3.860	32.97	11.53	97.18
ash	1	4.779	18.63	7.68	85.63
burn	3	4.341	17.64	23.35	66.70

path	1	4.368	27.29	12.42	86.79
mountain	1	4.780	19.47	14.14	79.35
red	4	5.055	17.11	10.66	82.64
green	4	5.510	17.09	12.66	68.67
yellow	4	5.501	20.06	9.32	68.74
white	4	4.834	18.95	17.67	88.99
black	4	4.968	20.10	15.08	88.56
night	1	4.815	24.27	13.30	91.91
hot	4	4.864	14.33	15.39	74.85
cold	4	5.101	16.35	13.55	84.75
full	4	5.025	19.77	15.01	65.03
new	4	4.786	27.87	9.59	80.44
good	4	4.551	14.33	15.82	90.40
round	4	6.066	15.53	17.62	53.64
dry	4	5.027	19.94	14.96	75.79
name	1	4.041	34.06	7.85	86.36
