# Supplementary material for: Cross-linguistic conditions on word length
Source: PLoS One. 2023 Jan 27;18(1):e0281041. doi: 10.1371/journal.pone.0281041 (PMC9882889; doi:10.1371/journal.pone.0281041)
Supplement: S9 File — (PDF) [file pone.0281041.s009.pdf]

S09: Why properties of items are estimated by averaging over WALS genera

Because of the geographic and phylogenetic distance between the languages assigned to the Eastern and Western Hemispheres, a positive correlation between hemispheres across items for a given property is unlikely to reflect shared history. Instead, the correlation is attributable to inherent differences between the items with respect to the property, and the strength of the correlation shows how well the property as measured can be generalized across samples. Table S09-1 presents the rank correlations between hemispheres for two measures of four properties.

Table S09-1. Spearman's rank correlations between hemispheres for measures of item properties.

	Language	Genus
Mean word length	.66	.76
Stability	.14	.73
Synonyms	.50	.62
Attestation	.31	.55

Both measures start with the mean for each item across doculects within languages as defined by ISO 639-3 codes. The Language measure then averages the language means across all languages. The Genus measure first averages the language means across languages within each WALS genus and then averages the genus means across all genera. The Genus measure produces higher correlations between hemispheres than the Language measure for all four properties: mean word length, stability, percent synonyms, and percent attestation. This improvement does not reflect a reduced variance with more inclusive units of analysis, because here the units of analysis (items) are the same in both the Language and Genus measures. Instead, the generalizability of the Language measure may be degraded by the undue influence of a few large genera such as Bantu and Oceanic, each of which is confined to a single hemisphere.
