# Supplementary material for: Cross-linguistic conditions on word length
Source: PLoS One. 2023 Jan 27;18(1):e0281041. doi: 10.1371/journal.pone.0281041 (PMC9882889; doi:10.1371/journal.pone.0281041)
Supplement: S10 File — (PDF) [file pone.0281041.s010.pdf]

S10: Morphology

Tables S10-1 – S10-8 contain data and results relating to the subsection Morphology of the section Results.

Table S10-1. Data on presence (1) / absence (0) of different word formation strategies (WFS) in the worldwide sample of [1], as well as mean word length (MWL). The columns representing WFS data are given in the same order as in the data sheet of Ref. [1, pp. 330-331], and (using the same abbreviations as in [1]) represent the WF categories PRX, SFX, IFX, CRX, PRX-SFX, PRX-IFX, IFX-SFX, RAP, VAL, PRX+VAL, SFX+VAL, CAL, CPN, NIN, RDP, CNV, BFR, BLN, STR, TOP.

Name	ISO-code	WFS	MWL
Amharic	amh	11100001000010100000	4.046
Anejom	aty	11000000100011101000	4.912
Bardi	bcj	01000000000010100000	5.289
Breton	bre	11000000001010010000	4.062
Cirecire	hio	01000000000010110101	3.859
Clallam	clm	11101001000011110000	5.612
Dangaleat	daa	01100000100000110001	3.500
Datooga	tcc	11001000100010110001	5.419
Diola-Fogny	dyo	01000000000000100000	4.411
English	eng	11001000101111111110	3.381
Estonian	ekk	11000000000011110100	3.806
Finnish	fin	11000000000010111100	4.356
Ga	gaa	11000000000010100000	3.150
Georgian	kat	11011000000010100100	4.575
Greek	ell	11000000000010011100	4.719
Hausa	hau	11000000101010110001	4.199
Hebrew	heb	11111111111011111110	4.075
Hungarian	hun	01000000000010010000	3.346
Ilocano	ilo	11111110000010110100	4.382
Indonesian	ind	11111000000010100000	4.731
Japanese	jpn	11101000000010110100	4.053
Jaqaru	jqr	01000000000010100000	4.446

Ket	ket	11000000100011010000	3.394
Konni	kma	1100100001101110000 1	3.962
Kwakwala	kwk	01000000000000110000	4.295
Lakhota	lkt	11001000000011000000	3.969
Luganda	lug	11100010101010100010	3.969
Luo	luo	11001000100010110000	3.700
Malayalam	mal	11000000001110000000	4.967
Mandarin_Chinese	cmn	1110010000001110000 1	2.844
Maori	mri	11000000000010110000	4.194
Marathi	mar	11001001111010111000	3.987
Movima	mzp	01000000000011100000	5.224
Nelemwa	nee	11011000001111110100	3.409
Pipil	ppl	11001000000011100000	3.778
Slavey	xsl	11000000000011000000	3.867
Slovak	slk	11111100101110111100	4.075
Spanish	spa	11110000000010011100	4.556
Swahili	swh	11001000000010110000	3.487
Tamil	tam	11010000000011101000	4.629
Tatar	tat	01100000000010100000	3.675
Telugu	tel	11000000000011110000	5.122
Tibetan	bod	01000000100010110000	3.201
Totonac	tku	11111000000011110000	5.050
Tzotzil	tzo	11000000100011100000	3.924
Udihe	ude	01000000000011110000	3.725
Vietnamese	vie	00000000000010110111	2.971
West_Greenlandic	kal	01000000000000000000	5.775
Wichi	mzh	11010000000011010000	4.494
Yoruba	yor	10100000000011110000	3.208
Zulu	zul	11001000000010110000	3.595

Table S10-2. Data on saturation in the European sample of [2] and mean word length (MWL)

Name	ISO-code	saturation	MWL
Abkhaz	abk	50	3.962
Adyghe	ady	42	3.850
Aghul	agx	16	3.500
Akwakh	akv	23	4.392
Albanian	sqi	44	3.797
Archi	arc	13	3.575
Avar	ava	42	3.665
Bashkir	bak	25	3.981
Basque	eus	49	4.469
Belorussian	bel	46	4.294
Bezhta	bez	37	3.350
Botlikh	bph	33	4.077
Breton	bre	37	4.062
Budugh	bdk	31	3.962
Bulgarian	bul	44	4.154
Catalan	cat	45	3.811
Chuvash	chv	33	3.831
Crimean Tatar	crh	29	3.628
Croatian	hrv	49	4.112
Czech	ces	50	4.200
Danish	dan	35	4.025
Dargwa	dar	28	3.714
Dutch	nld	44	3.625
English	eng	50	3.381
Estonian	ekk	38	3.806
Faorese	fao	43	4.125
Finnish	fin	36	4.356
French	fra	42	3.025
Frysian	frs	35	3.586
Gagauz	gag	30	4.227

German	deu	49	4.025
Irish	gle	36	3.631
Greek	ell	37	4.719
Hungarian	hun	24	3.346
Icelandic	isl	38	4.419
Italian	ita	37	4.771
Kabardian	kbd	43	3.763
Kalmyk	xal	42	4.088
Karaim	kdr	22	3.406
Kashubian	csb	36	4.100
Khinalug	kjj	22	3.423
Khwarshi	khv	30	3.425
Komi	kpv	24	3.319
Ladin	lld	30	3.858
Lak	lbe	27	3.929
Latvian	lvs	34	4.544
Lithuanian	lit	35	5.319
Makedonian	mkd	42	3.981
Maltese	mlt	45	4.487
Mari	mhr	39	3.754
Mordvin	myv	17	4.062
Nenets	yrk	19	3.925
Norwegian	nor	48	3.535
Ossetic	oss	21	3.908
Polish	pol	47	4.419
Portuguese	por	46	4.075
Romanian	ron	49	4.158
Russian	rus	46	4.231
Rutul	rut	21	3.885
Sardinian	sro,src	27	4.706
Serbian	hbs	47	4.000
Slovak	slk	50	4.075

Slovenian	slv	44	4.119
Spanish	spa	43	4.556
Swedish	swe	46	3.504
Tat	jdt	32	3.925
Tatar	tat	31	3.675
Turkish	tur	33	4.088
Udi	udi	25	3.658
Udmurt	udm	22	3.538
Ukrainian	ukr	46	4.463
Upper Sorbian	hsb	38	4.112
Welsh	cym	31	4.075

Table S10-3. Results of two-sided t-tests of a difference in MWL between languages with and without a particular WFS (*N* = 51).

WFS	t(49)	p
PRX	0.075	0.941
SFX	2.281	0.027
IFX	-0.111	0.912
CRX	1.293	0.202
PRX-SFX	0.266	0.791
PRX-IFX	-0.900	0.373
IFX-SFX	-0.007	0.995
RAP	0.854	0.398
VAL	-0.992	0.326
PRX+VAL	-0.348	0.729
SFX+VAL	-0.687	0.495
CAL	-0.556	0.581
CPN	1.053	0.298
NIN	0.099	0.922
RDP	-0.862	0.393
CNV	-1.352	0.182
BFR	0.731	0.468
BLN	-0.766	0.447
STR	-1.665	0.102
TOP	-1.333	0.189

Table S10-4. Correlations between mean word length, log inventory, and log population across macroareas (*r* in lower triangle, *p* in upper triangle) using all 40 words (cf. Table 1 of main text).

	Word length	Log inventory	Log population
Word length		0.1994	0.0087
Log inventory	-0.61		0.1123
Log population	-0.92	0.71	

Table S10-5. Correlations between mean word length, log inventory, and log population across macroareas (*r* in lower triangle, *p* in upper triangle) using only the five shortest words.

	Word length	Log inventory	Log population
Word length		0.4221	0.0364
Log inventory	-0.41		0.1123
Log population	-0.84	0.71	

Table S10-6. Correlations between mean word length, log inventory, and log population across languages (*r* in lower triangle) using all 40 words (cf. Table 1 of main text).

	Word length	Log inventory	Log population
Word length			
Log inventory	-0.37		
Log population	-0.34	0.36	

Table S10-7. Correlations between mean word length, log inventory, and log population across languages (*r* in lower triangle) using only the five shortest words.

	Word length	Log inventory	Log population
Word length			
Log inventory	-0.29		
Log population	-0.23	0.36	

Table S10-8. Mean word length for different macroareas

MWL	all forty	five shortest
Africa	3.674	2.964
Eurasia	3.891	3.249
NG and Oceania	4.095	3.091
N America	4.189	3.448
S America	4.433	3.551
Australia	5.234	4.450

References

1. Štekauer P, Valera S, Körtvélyessy L. Word formation in the world’s languages: a typological survey. Cambridge: Cambridge University Press; 2012.

2. Körtvélyessy L, Štekauer P, Genči J, Zimmermann J. Word-formation in European languages. Word Struct 2018;11:313–358.
